# Specificity Rendering ‘Hot-Spots’ for Aurora Kinase Inhibitor Design: The Role of Non-Covalent Interactions and Conformational Transitions

**DOI:** 10.1371/journal.pone.0113773

**Published:** 2014-12-08

**Authors:** Preethi Badrinarayan, G. Narahari Sastry

**Affiliations:** Molecular Modeling Group, Organic Chemical Sciences, CSIR-Indian Institute of Chemical Technology, Tarnaka, Hyderabad- 500 607, India; Hong Kong University of Science and Technology, Hong Kong

## Abstract

The present study examines the conformational transitions occurring among the major structural motifs of Aurora kinase (AK) concomitant with the DFG-flip and deciphers the role of non-covalent interactions in rendering specificity. Multiple sequence alignment, docking and structural analysis of a repertoire of 56 crystal structures of AK from Protein Data Bank (PDB) has been carried out. The crystal structures were systematically categorized based on the conformational disposition of the DFG-loop [in (D_I_) 42, out (D_O_) 5 and out-up (D_OU_) 9], G-loop [extended (G_E_) 53 and folded (G_F_) 3] and αC-helix [in (C_I_) 42 and out (C_O_) 14]. The overlapping subsets on categorization show the inter-dependency among structural motifs. Therefore, the four distinct possibilities a) 2W1C (D_I_, C_I_, G_E_) b) 3E5A (D_I_, C_I_, G_F_) c) 3DJ6 (D_I_, C_O_, G_F_) d) 3UNZ (D_OU_, C_O_, G_F_) along with their co-crystals and apo-forms were subjected to molecular dynamics simulations of 40 ns each to evaluate the variations of individual residues and their impact on forming interactions. The non-covalent interactions formed by the 157 AK co-crystals with different regions of the binding site were initially studied with the docked complexes and structure interaction fingerprints. The frequency of the most prominent interactions was gauged in the AK inhibitors from PDB and the four representative conformations during 40 ns. Based on this study, seven major non-covalent interactions and their complementary sites in AK capable of rendering specificity have been prioritized for the design of different classes of inhibitors.

## Introduction

Aurora kinase (AK) is a serine-threonine protein kinase located in the nucleus and is involved in the regulation of cell division [1], [2]. The three of its isoforms A, B and C have different substrate specificities and function. The A and B isoforms are expressed in proliferating cells whereas the C isoform is usually expressed in germ cells. Aurora A and B isoforms are thus involved in mitosis and are associated with cancer [3], [4]. This has resulted in a number of potent candidates such as VX680, AT9283, ZM-447439, Hesperadin, and MLN8237 which are now in clinical trials [5]–[9]. Majority of the aforementioned inhibitors target the conserved ATP site in the DFG(Asp-Phe-Gly)-in conformation or explore the allosteric site exposed through the classic DFG-flip [10]–[15]. However, there are some inhibitors which target an unusual non DFG-out conformation called DFG-out (up) conformation which is formed through ligand-induced conformational changes and results in switching the character of the active site from polar to hydrophobic [16]–[19]. This conformation is formed when the DFG-loop is ushered to a location parallel to the αC-helix unlike the regular DFG-out wherein it swaps out of the active site [20]. The type I inhibitors targeting the DFG-in conformation are less target specific due to the conserved nature of the active site to which they bind. The type II inhibitors binding to the DFG-out conformation are known to cause side-effects and are prone to resistance [21]. These varied kinase conformations are formed due to the transition of the DFG-loop [22], [23]. Therefore, targeting the DFG-out conformation is advantageous to achieve specificity and overcome resistance.

The DFG-flip is accompanied by a series of conformational changes which alters the arrangement of the major structural motifs in a co-ordinated fashion [24], [25]. Studies of kinase crystal structures and MD simulations have shown that the structural motifs such as the DFG-loop, αC-helix, Glycine rich loop (G-loop) and the activation loop (A-loop) form varied inactive conformations on transition [26]–[28]. With each conformational variation, the interaction-networks formed by the major residues of the structural motifs get disrupted and re-engineered [29]. The interaction-networks are made up of a closely knit circuit of non-covalent interactions [30]–[33]. Several inhibitors have been designed which use a specific non-covalent interaction in addition to hydrogen bond (H-bond) to achieve specificity [34]–[36]. The AK inhibitor VX680 and the p38 MAP kinase inhibitor SB203580 achieve specificity by forming π-π stacking interaction with the aromatic residue (Tyr or Phe) in the G-loop signature sequence HGXGX(Y/F)GXVH [19], [37], [38]. Similarly, to obtain specificity through interactions, Soliva et al. added a sulfonyl phenyl moiety to the pyridinyl heterocycle core and Laufer et al. designed 2-thioimidazole derivatives while Natarajan et al. introduced a phthalimide group to the 3,4-dihydropyrido [4,3-d]pyrimidazin-2-one template [39]–[41]. Dasatinib obtains specificity for Bruton's tyrosine kinase through cation-π interaction formed by its *ortho*-chloro methyl phenyl ring with the ε-amino group of the salt-bridge former Lys430 [42]. Whereas in 4-(phenylamino)-pyrrolo [2,1-f] [1], [2], [4] triazine, the methyl hydroxamate begets specificity through a CH-π interaction with the DFG-loop Phe169 [43]. The set of non-covalent interactions that can be formed by an inhibitor with different regions of the active site varies with conformation [44]–[47]. Several of these binding site regions also referred as 'hot-spots' when targeted by an inhibitor are capable of rendering specificity [48]. Hence, it would be useful to prioritize the non-covalent interactions complementing these specific sites. The conformational transitions have a profound effect on the binding site character and non-covalent interactions. While the influence of transition on the topology of binding is well studied, there is a very primitive understanding about its impact on the nature of non-covalent interactions and their role in achieving specificity. We have therefore identified distinct active and inactive conformations of the three structural motifs namely the DFG-loop, αC-helix and G-loop. These conformations have been used to study the impact of conformational transitions on the individual residues of these motifs and on the participation of various non-covalent interactions. The insights obtained have been used to prioritize seven non-covalent interactions which complement the binding of different inhibitors and can prove useful in achieving specificity.

## Materials and Methods

### 1. Structure- Sequence Analysis

56 crystal structures of AK from PDB and their respective sequences from UNIPROT were downloaded and used as start-ups [49], [50]. Three organisms namely *Homo sapiens*, *Xenopus laevis* and *Mus musculus* contribute to the 56 crystal structures (H: 43, X: 5, M: 8) and their 157 co-crystals (H: 113, X: 8, M: 36). The structures were individually analysed in detail in terms of quality and sequence. The resolution of these crystal structures is in between 1.60 to 3.35 Å. Among them, 22 structures have different types of modified residues. Herein, the threonine was modified to phosphothreonine (TPO: 18); metheonine into selenomethionine (MSE: 1), tyrosine into *O*-phosphotyrosine (PTR: 2) and cysteine into S, S-(2-hydroxyethyl) thiocysteine (CME: 4) (Table S1 in File S2). The structures are made of sequences differing in length and constituting both homo- and hetero-chains of AK A and B isoforms (Table S2 in File S2). Majority of the structures comprise of the O14965 (AURKA_HUMAN). A pairwise sequence alignment was done using blast-p to identify kinases which are sequentially similar to AK [51]. The AK sequence AURKA_HUMAN was used as the reference sequence against the entire kinome present in kinbase v1.1. [52] The kinases having a sequence identify of more than 30% were filtered in (Table S3 in File S2). Since the focus was on identifying specificity determinants among human kinome, the kinase domains of only *Homo sapiens* were retrieved from the filtered sequences. A multiple sequence alignment was constructed (MSA) from these sequences to observe the conservation pattern and to identify the unique residues which can be targeted to obtain specificity through binding. The MSA with ClustalW was constructed using a Gonnet matrix with a gap open and gap extension penalty of 10 and 0.1 for pairwise alignment [53]. In addition, a gap distance of 5 was set to build the tree using Neighbour-Joining method. The MSA was used to identify the possible sites for target specific inhibitor binding in AK based on conservation. A kinase signature profile was generated to validate the uniqueness of the residues identified through MSA [54].

### 2. Docking

The downloaded AK crystal structures and their co-crystals were subjected to docking in order to evaluate the binding interactions of the inhibitors and to prioritize the binding-site residues forming interactions. The breaks in the crystal structures were modelled with Modeller 9v5 and side-chains were refined using Prime module of Schrodinger 8.0 [55], [56]. In majority of the crystal structures, the breaks are not in the core binding site. 3E5A/O14965 was used as template to model the missing residues in the DFG-in conformation structures while 2J4Z/O14965 was used for DFG-out structures. The quality of the modelled protein was cross validated with Ramchandran plot (RAMPAGE), energy profiles with ProSA and the secondary structure was determined with STRIDE [57]–[59]. The protein preparation wizard was used to prepare the proteins after adding hydrogen. Ligands were submitted to the LigPrep module to generate a range of ionization states populated at a given pH range of 7.4±2 followed by an exhaustive conformational sampling with Confgen [60]. The rigid docking protocol Glide 4.5 has been employed. The two options of docking namely standard precision (SP) and extra precision (XP) of ‘Glide’ module were used for docking the generated conformers of the co-crystals to their respective receptors [61]. The grid was generated by specifying the co-crystal as grid centre. The default SP docking settings were used and the conformations obtained from SP were used as input for XP. Hydrophobic and hydrophilic maps were generated to probe the solvent accessible regions. Ten poses were retained for each of the docked co-crystal in both the docking protocols. The docked-complexes were used to investigate the binding of inhibitors to the two DFG-conformations.

### 3. Molecular Dynamics

Six distinct conformations of AK structural motifs bound to different inhibitors were selected as representatives based on a cluster analysis and subjected to MD simulations for 40 ns with Desmond along with two apo proteins (Table 1) [62]. Systems I, II, IV-VI comprise of the O14965 sequence while system III is made up of P97477. The apo DFG-in form was generated from PDB: 1OL5 and apo DFG-out from PDB: 3UO6 due to the absence of an apo crystal structure. The phosphate groups of pThr287 and pThr288 were removed and simulated [24]. The ligand parameters were generated with the Schrodinger software. The MD simulations were performed using the OPLS 2005 force field and TIP3P model [63], [64]. All systems were solvated in an orthorhombic water-box with a 10 Å buffer region between the solute structures and the simulation box boundary on each side. The systems were initially equilibrated using default protocol employed in Desmond. This comprises of a series of restrained minimizations and MD simulations which are designed to slowly relax the system without any substantial deviation from the initial protein co-ordinates. All MD simulations were conducted at constant pressure (1 bar) and temperature (300 K) maintained using Berendsen barostat and thermostat algorithms respectively. The pressure and temperature control used a relaxation time of 0.5 ps. RESPA integrator was used for all simulations [65] with a 2.0 fs time step for bonded, van der Waals and short-range Coulomb interactions and a 6.0 fs time step for long-range Coulomb interactions. The production run was carried out for 40 ns using the isothermic-isobaric ensemble (NPT) and frames were recorded at an interval of 1.2 ps. The trajectories were sampled for conformational variants. The trajectories were used to understand the conformational variations and their influence on the nature of inhibitor binding interactions. The trajectories obtained on simulation of apo proteins were used as reference to monitor the changes during simulation in other four inhibitor bound conformations. An ensemble of 4000 snapshots was extracted from systems II and IV and was used as a test-set to evaluate the performance of the developed metric (S1 Figure in S1 File).

**Table 1:** Details of the systems subjected to MD simulations.

System	Structure	Inhibitor	DFG-Loop	αC-helix	G-loop
I	2W1C	LOC	DFG-in (D_I_)	αC-helix in (C_I_)	Extended (G_E_)
II	3E5A	VX6	DFG-in (D_I_)	αC-helix in (C_I_)	Folded (G_F_)
III	3DJ6	AK6	DFG-in (D_I_)	αC-helix out (C_O_)	Folded (G_F_)
IV	3UNZ	OBZ	DFG-out up (D_OU_)	αC-helix out (C_O_)	Extended (G_E_)
V	Apo	-	DFG-in (D_I_)	αC-helix in (C_I_)	Extended (G_E_)
VI	Apo	-	DFG-out up (D_OU_)	αC-helix out (C_O_)	Extended (G_E_)

### 4. Development of metric to gauge kinase conformations

To understand the impact of conformational variations on the nature of participating interactions it is essential to distinguish them. Therefore, a metric based on the pairwise distances and angles of key residues of AK structural motifs was developed to gauge the inter- and intra-motif variations in the studied DFG-in and the DFG-out (up) conformations of AK. Four key residues identified through the structural analysis and MD simulations were used to develop parameters for the inter-motif metric. The conserved residue and salt-bridge former Lys162, the gatekeeper (GK) Leu210 at the mouth of the hinge, the DFG-loop Phe275 and the salt-bridge former Glu181 of αC-helix were used to develop the parameters for the inter-motif metric. Different measures such as α-carbon, volume, summation and centre of mass (COM) were considered to map the variations among the selected set of amino acid residues [66]–[69] (S2 Figure in S1 File). Among them COM displayed highest precision in discriminating the kinase conformations. Nine parameters, four distance parameters r2(GK⋯E181), r3(GK⋯F275), r4(K162⋯F275), r6(K162⋯E181) and three angle parameters ∡K(GK+2)E, ∡K(GK+2)F and ∡E(GK)F were used in the inter-motif metric (S4 Table in S2 File, S3 Figure in S1 File). The contribution of each individual parameter of the inter-motif metric showed that five parameters consisting of the four distance (r2, r3, r4, r6) and one angle parameter ∡E(GK)F clearly distinguish the DFG-in and DFG-out (up) conformations (S4a Figure in S1 File). The statistical analysis has been done using XLSTAT 2012 [70]. Different regression models were generated with different variables taking into account the prediction accuracy and % Error. The metric has been tested with a test set comprising of AK crystal structures from PDB followed by an ensemble of 4000 structures extracted from the MD trajectories (S5 Table, S6 Table in S2 File). The performance of the test set reiterated the pattern observed during the development of parameters (S4b Figure in S1 File). Six distance parameters r1(A273⋯D274), r2(D274⋯F275), r3(F275⋯G276), r4(D274⋯G276), r5(F275⋯W277), r6(F275⋯T288) and three angle parameters ∡DFG, ∡FWH, ∡FWT were used to measure the spread of the DFG-loop and the conjoin A-loop (S5 Figure in S1 File). Among them, four parameters namely the two distance parameters (r5, r6) and the two angle parameters ∡DFG, ∡FWH were precise in distinguishing the DFG-conformations (S7 Table in S2 File, S6a Figure in S1 File). The intra-motif metric was tested with same test set as the inter-motif metric (S8 Table, S9 Table in S2 File). The performance of the test set again reiterated the pattern observed during the development of parameters (S6b Figure in S1 File). Both the metric discriminate the three conformations among the reported crystals and ensembles obtained from MD simulation advocating their precision in identifying the kinase conformation.

### 5. Interaction analysis

The kinase inhibitor complexes were clustered into 40 bins on the basis of similarity using maximum dissimilarity algorithm. Cluster centres representing each cluster were identified and further segregated into the four different classes of kinase inhibitors namely type I, type II, type I^1^/_2_ and type III. Pharmacophore mapping was carried out using Phase module of Schrodinger 8.0 [71]. Pharmacophore features were identified through maximum common subgraph from a set of 3D-molecular graphs of kinase inhibitors. The pharmacophore generated for the four inhibitor classes were aligned with the active site to map the drug-receptor interactions of each class of inhibitors. The pharmacophore interactions were then correlated with the structural motifs. The interactions were analysed with the docked complexes and MD trajectories using the criterion given by Rognan et al. [72]. Structure interaction fingerprints encoding the presence or absence of interactions of a certain feature of the inhibitor with the amino acid residues of the AK binding site were generated from the docked complexes. The H-bond, van der Waals, salt-bridge, cation-π and π-π analysis was done using in-house scripts [73]–[75]. The interactions were keyed into a fourteen bit binary vector with each bit representing a specific interaction of the inhibitor with a particular residue of the AK binding site.

## Results and Discussion

### 1. Specificity determinants in AK sequence

**Figure 1:**
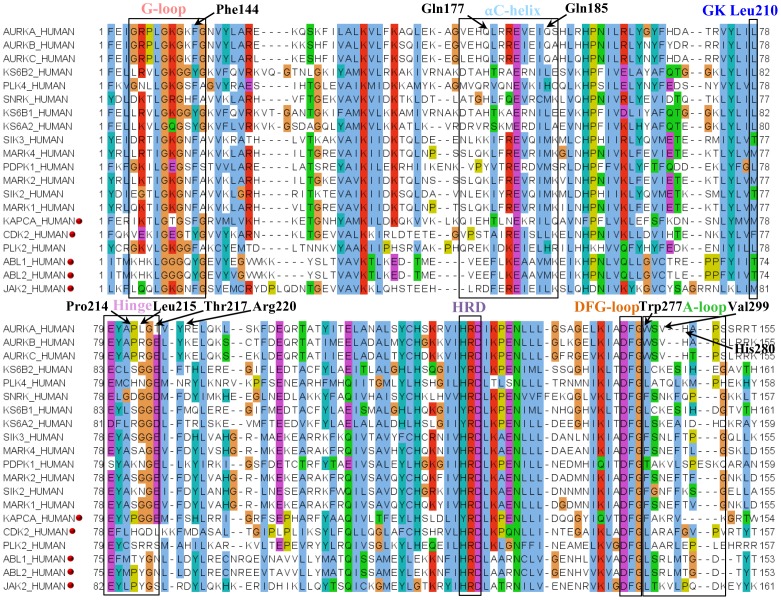
MSA of the kinase domains of AK and other sequentially similar kinases obtained from kinbase. A pairwise sequence alignment of the AK sequence (AURKA_HUMAN) against the annotated kinome present in Kinbase v1.1 was done using blast-p (Table S3 in File S2). The kinase domains of the sequentially similar sequences were retrieved from Kinbase v1.1 and a MSA was constructed with ClustalW 2.1. Jalview 2.8.0 has been used to view the alignment and the colour scheme is as per ClustalX. The red dots represent kinases which have been crystallized with different AK inhibitors (Table 2). The labelled residues indicate the possible sites for target specific inhibitor binding in AK based on conservation.

The MSA shows high sequence similarity in the catalytic domains across the species (Fig. 1). The three AK isoforms share a high sequence similarity of more than 70% in their kinase domain. The major structural motifs such as G-loop, αC-helix, hinge, DFG-loop and A-loop comprise of residues which are specifically found in AK. The residues Glu175, His176, Gln177, Leu178, Ile182, Glu183, Gln185 of αC-helix are unique. Of these Gln185 occupies the conserved hydrophobic R-spine residue position in αC-helix and therefore its uniqueness can be easily enhanced to beget specificity. In the DFG-out(up) structures Gln185 is at the centre of a network of polar interactions formed by Asp274, Arg255 and Leu194. Dodson et al. have also shown that 53% of kinases have Leu at this position and 25% have Met [20]. In the DFG-in structures, these residues interact with side-chain of the DFG-loop Phe. The AK has a small sized Leu210 as the GK while its sequentially close neighbours have a larger sized gatekeeper. The tail end of the hinge residues Pro214 (GK+4), Leu215 (GK+5), Thr217 (GK+7) and the αD-helix Arg220 (GK+10) are non-conserved. Thus, a H-bond at this position of the ATP-pocket would not only contribute to tight binding resulting in improved efficacy but will also contribute to specificity. Among these hinge residues, Leu215, Thr217 and Arg220 are found only in Aurora A isoform and can thus also be used to achieve isoform selectivity. While Leu is substituted with Arg in Aurora B and C, Thr is substituted with Glu and Arg is substituted with Lys. In a recent study, Bavetsias et al. have also identified the role of Thr217 in eliciting Aurora A isoform specificity [76]. The A-loop contains three unique residues Trp277 (D+3), Val279 (D+5) and His280 (D+6). Phe144 is the aromatic residue in the key –GxGxFG- motif of the G-loop in AK and most of its neighbours. However, Lys143, Glys145 and the preceding Gly136, Arg137, Pro138, Leu139 that form the intra H-bonds between the two strands of β2 are specific to AK. This might be a reason for the extra plasticity of the G-loop of AK which enables it to enter the binding site and fold. The G-loop folding changes the position of the aromatic residue Phe144 and facilitates its interaction with the inhibitor. The kinase signature profile validates the uniqueness of the aforementioned residues identified through sequence alignment (S7 Figure in S1 File).

### 2. Conformational space of AK crystal structures

**Figure 2:**
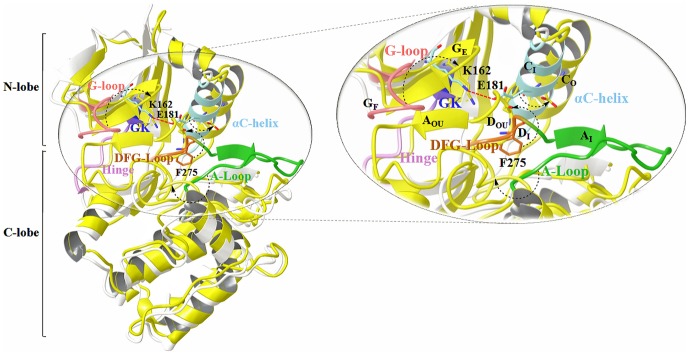
Conformational variations in the major structural motifs of AK concomitant with DFG-flip. In the figure, the two conformations of AK, active DFG-in (PDB: 3E5A, white) and the inactive DFG-out (up) conformation (PDB: 3UNZ, yellow) are superimposed. The major structural motifs of DFG-in are multi-coloured while that of DFG-out (up) are depicted in yellow. The figure displays the key components of the AK kinase active site: G-loop, αC-helix, gatekeeper (GK) hinge, DFG-loop, A-loop; and their critical residues: salt bridge formers Lys162, Glu181 (αC-helix) and Phe275 (DFG-loop). The arrows depict the differences in the two conformations resulting due to the DFG- and A-loop flip, αC-helix rotation and G-loop folding. The G-loop is in the folded conformation (G_F_), αC-helix is in the 'in' conformation (C_I_) and A-loop in 'in' conformation (A_I_) in the DFG-in structure shown in figure while in the DFG-out (up) structure, the G-loop is in the extended form (G_E_), αC-helix is in the 'out' conformation (C_O_) and A-loop in 'out (up)' conformation (A_OU_). The co-crystals have been removed for clarity.

**Figure 3:**
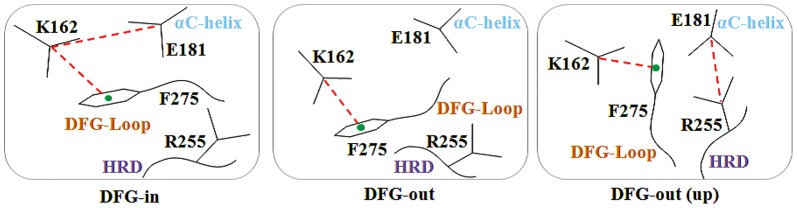
The three major DFG-loop conformations observed in AK. The figure displays the synergy between the salt-bridge and cation-π interactions in different DFG-conformations of AK. The interacting partners are the conserved Lys162 (β3), Glu181 (αC-helix), Phe275 (DFG-loop) and Arg255 (HRD motif, the conserved triad found in the catalytic loop of most kinases).

The movement of the major structural motifs such as the DFG-flip, αC-helix rotation and G-loop folding cause varied changes in the active and inactive conformations of kinases (Fig. 2) [77]–[79]. The DFG-flip from the active DFG-in to an inactive 'DFG-out' or 'DFG-out (up)' conformation unravels the non-conserved hydrophobic allosteric site [20]. The αC-helix rotation to the 'αC-helix out' conformation displaces the conserved Glu and causes a disruption of the conserved Lys-Glu salt bridge [17]. Guimaraes et al. and Doerksen et al. have demonstrated that even the usual extended G-loop in kinases displays a tendency to form a caged or folded conformation [38], [80]. These conformational changes are known to vary with the sequence composition of the target kinase and nature of the bound inhibitor. Therefore, in order to study the variations of the motifs and specificity determinants identified through the sequence analysis, we have used a set of distance and angle measures between conserved motifs in these segments to classify the 56 inhibitor bound conformations of AK. The AK crystal structures were grouped into three bins namely DFG-in (42), DFG-out (up) (9) and DFG-out (5) using the inter- and intra-motif metrics variables. The crystal structures illustrate the presence of two major types of non DFG-in conformations formed as a result of the DFG-flip (Fig. 3). In majority of the non DFG-in conformations of AK structures, the DFG-loop is turned upward with Phe275 placed between the two salt-bridge formers. As compared to the DFG-in structures, in non DFG-in structures, the side-chain of Asp274 in the DFG-out (up) is rotated around 180° and lies parallel to the αC-helix whereas the Phe275 lies above Glu181. A minor set however represents the regular DFG-out conformation bound by type II inhibitors. In these structures, the DFG-loop is observed to have moved from the N-lobe as seen in DFG-in structures to the C-lobe with Asp274 placed downwards. In this case, the Asp274 and Phe275 lie on opposite sides. Comparative analysis of the structures in the two conformations shows that the positions of the residues Ala273, Asp274, Phe275, Gly276 and Lys271 vary (S8a Figure in S1 File). As compared to the DFG-in conformation, the residues Ala273, Asp274, Phe275, Trp277 and His280 are available for inhibitor binding in the non DFG-in structures, especially in the DFG-out (up). Among them, Trp277 and His280 are sequentially unique residues.

The αC-helix rotation and DFG-loop flip was found to be complementary in the AK structures. Considering the nature of a standard right handed α-helix, the ‘*I*’, ‘*i+4*’ and ‘*i-4*’ distances and dihedral angles were studied to understand the conformational differences in αC-helix. In addition, three distance variables r4(K162⋯F275), r6(K162⋯E181), ∡E(GK)F were also used. The two glutamines (Gln177: E-4 and Gln185: E+4) positioned four residues away on either side of Glu181 vary substantially in the active and inactive conformations (S8b Figure in S1 File). Of these, Glu185 is an important residue of the R-spine and is sequentially non-conserved. Although substantial conformational rearrangement is observed in the DFG-loop and αC-helix of the inactive conformation structures as compared to the DFG-in structures, the distance between Phe275 of DFG-loop and Glu181 of αC-helix r5(E181⋯F275) does not show much difference. The Phe275 lies below Glu181 in DFG-in conformation while in non DFG-in structures, it lies adjacent to it. The distance of the peptide backbone dihedral angles (φ = −140°, ψ = 135°) of the anti-parallel β1 and β2 sheet harbouring the G-loop were used to gauge variations in the G-loop of the AK crystal structures. The folded G-loop conformation was observed only in three of the AK structures namely PDB: 3DJ5, 3DJ6, 3E5A. Comparative arrangement of the G-loop residues showed positional variation of Leu139, Lys141, Gly142, Lys143, Gly145 and Phe144 in the extended and folded conformations (S8c Figure in S1 File). Among these, the hydrophobic residue Leu139 is known to render stability to the receptor while Phe144 participates in ligand binding [27]. The structure analysis shows variations in the positions of binding site residues in different conformations which in turn affects their availability for inhibitor binding. It has also been deciphered through sequence analysis that many of these residues are non-conserved and can be used as specificity determinants. Therefore, to gain insights on the participation of various residues and the nature of inhibitor binding interactions in different AK conformations, six distinct conformations of AK structural motifs were selected based on the structure analysis and subjected to MD simulations (Table 1, S9 Figure in S1 File).

### 3. Impact of conformational variations on the nature of non-covalent interactions

**Figure 4:**
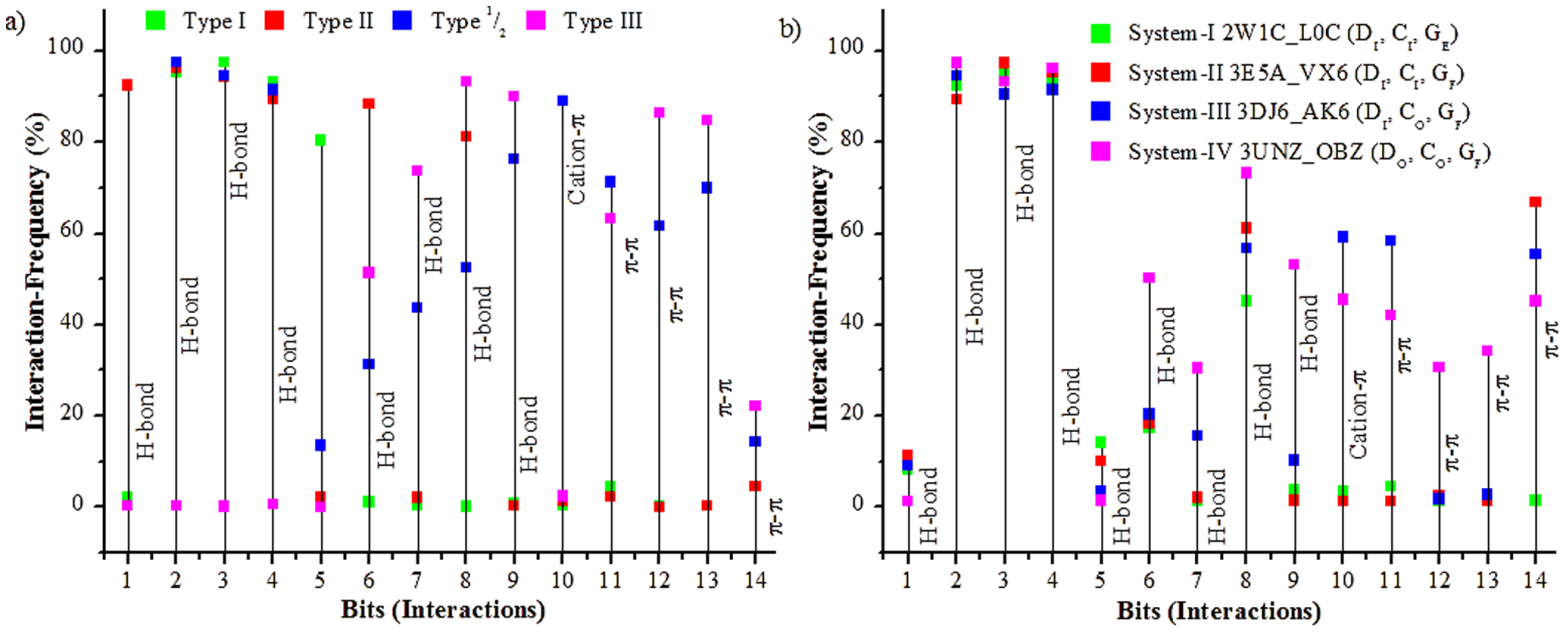
Major interaction sites in AK. The graphs depict the frequency of the most prominent interactions with different regions of the binding site formed by a) different classes of AK inhibitors from PDB and b) by the inhibitors bound to the four representative conformations during the 40 ns MD simulation. The four classes of inhibitors depicted in fig. 4a are the type I inhibitors which bind to the conserved ATP site in the DFG-in conformation, the type I^1^/_2_ which explore an additional back-pocket (BP) formed by the GK in addition to the ATP site in the DFG-in conformation, type III which bind to the allosteric pocket (HPII) in the DFG-out conformation and the type II which explore both the ATP and allosteric pockets in the DFG-out conformation. The details of the simulated systems and inhibitors in fig. 4b have been given in Table 1. The legend 4b describes the system, PDB id of the starting structure, its bound inhibitor and conformation of the major structural motifs. The x-axis represents interactions formed by different pharmacophore and their complementary sites in the binding pockets as given in Table 3.

The MD trajectories showed that each structural motif harbours key residues which consistently participate in protein-ligand interactions. The four inhibitors bound to the different AK conformations in systems I-IV accessed different sets of residues of the binding-site through different non-covalent interactions during MD simulations. Therefore, the docked receptor-inhibitor complexes from PDB and the frames extracted from the MD simulation were used to map the interactions and identify their complementary sites. The docked complexes were used to study the binding modes of inhibitors. The scoring components of the docked receptor-inhibitor complexes of AK showed major participation of H-bond and van der Waal's interactions (S10 Table in S3 File). Analysis of the docked poses show that the inhibitors formed cation-π, CH-π, π-π, salt-bridge and H-bond interactions with different binding site residues (Table 2). The major interactions formed were keyed into a 14 bit vector wherein each bit represents a non-covalent interaction (Table 3). The interaction-fingerprints thus obtained were used to correlate different pharmacophore features of the docked inhibitor complexes and non-covalent interactions. H-bond was found to be one of the most important interactions between AK and its inhibitors (Fig. 4a, S11 Table in S2 File). The conserved hinge H-bonds were the most prominent of all interactions. The backbone amide nitrogen of Ala213 forms H-bond with the acceptor atom of the inhibitor in over 80% of AK inhibitor docked complexes with a median average distance of 2.5 Å. The backbone carbonyl oxygen atom of Glu211formed a H-bond with the acceptor atom of the inhibitor with a median distance of 2.6 Å in 40% of the docked complexes. In most of the docked complexes, Leu210, Glu181 and His280 interacted with the donor atom of the inhibitor while Gly276 and Lys162 interacted with the acceptor atom of the inhibitor forming strong H-bonds like N-H⋯O, O-H⋯O and N-H⋯N. In around 70% of the non DFG-in docked complexes, the acidic side-chain of Asp274 formed H-bond with the donor atom of the inhibitor. The planar conformations of the linked heterocyclic systems found in type I and type II AK inhibitors of the docked complexes formed weak H-bonds like C-H⋯O, C-H⋯N. Gly276 and Glu181 in most cases acted as well established H-bond donor and acceptor respectively. The Glu181 in αC-helix out conformation forms H-bond with the linker connecting the ATP and allosteric sites. On comparative analysis of the docked complexes and MD trajectories, it was observed that the distance between the gatekeeper (GK) and DFG-loop Phe275 (r3(GK⋯F275)) as well as that between Lys162-Phe275 (r4(K162⋯F275)) is higher in non DFG-in conformation as compared to the DFG-in conformation. This difference in distance among key residues is observed to provide room for the binding of large heterocyclic systems to the non DFG-in structures. As compared to the DFG-in structures, the DFG-loop Phe275 in the DFG-out (up) is placed in a hydrophobic milieu whereas the Asp274 is surrounded by polar residues. The type II inhibitors were seen to bind to the DFG-loop Asp274 in the non DFG-in structures while the heterocyclic systems bind to Phe275 through π-π interactions. Analysis of the docked complexes and the MD trajectories show that the cation-π and ion-pair interactions co-exist among the key residues in different AK conformations. The disruption of one interaction is observed to facilitate formation of other. For example the ion-pair interaction among the conserved Lys162 and Glu181 observed in the DFG-in structures is not present in the non DFG-in structures. In these structures, Glu181 points away from the conserved Lys162 since here αC-helix is rotated out. However in these structures, Lys162 was seen to form cation-π interactions with the aromatic motifs of both DFG-loop Phe275 and inhibitor. Likewise the Glu181 engages in an ion-pair interaction with Arg255 of the HRD motif of the catalytic loop in the DFG-out (up) conformation (Fig. 3). Thus, a possible synergy is seen among the salt-bridge and cation-π interactions in different DFG-conformations of AK. The relative effect of one interaction over the other is an interesting phenomenon which has been studied by us in several systems and its exploration might unravel the cooperativity in kinases [81], [82]. In the DFG-out (up) conformation, docking analysis showed that the A-loop residues such as Trp277 and His280 formed π-π interactions with the aromatic rings of the type II inhibitors. The aromatic residue Phe144 of the G-loop operates independent of the DFG-loop conformation. The inhibitors bind to the aromatic motif of Phe144 through π-π or CH-π interaction. The frequency of these major interactions observed in the docked complexes was also studied with the MD trajectories to affirm their consistency over a period of time (Fig. 4b).

**Table 2:** Amino acid residues of AK which form important non-covalent interactions required for the conformational stability of the receptor and binding of inhibitors.

S No	Non-covalent interactions	G-loop	DFG-loop	A-loop	αC-helix	Hinge
1.	**π-systems:**	F144^G-loop^	F275^DFG-loop^	W277^A-loop^	Y246	Y212^Hinge^
	Aromatic residues	Y148^G-loop^	F144^G-loop^	F275^DFG-loop^	F165	F144^G-loop^
	(Phe, Tyr, Trp)	F275^DFG-loop^	W277^A-loop^	Y246	Y199	Y197
			Y246		Y207	Y246
					F200	
2.	**Cationic-systems:**	R195	K162	R255^HRD^	K166 ^αC-helix^	R195
	Positively charged R-groups	R189		R180^HRD^	R179^ αC-helix^	R189
	(Lys, Arg)				R205	R137
					R189	
3.	**Salt-bridge:**	E211^Hinge^	-	E260	E181^αC-helix^	E211^Hinge^
	Anionic carboxylate (RCOO^-^)	E260				
	Negatively charged R-groups					
	(Asp, Glu)					
	Cationic ammonium (RNH_3_^+^)	K143^G-loop^		R285^A-loop^	K162	K145^G-loop^
	of Lys and guanidinium	K141^G-loop^		K309	R195	K141^G-loop^
	(RNHC(NH_2_)_2_^+^) of Arg					K271

**Table 3:** Features of the 14 bit-vector used to deduce the interaction fingerprints.

Bit No.	Residue : Pharmacophore	Interaction
1	GK, Leu210 : HD	H-bond
2	GK+1, Glu211 : HD	H-bond
3	GK+2, Tyr212 : HA	H-bond
4	GK+3, Ala213 : HD	H-bond
5	GK+6, Gly216 : HD	H-bond
6	Ala273 : HD	H-bond
7	αC-helix, Glu181 : HD	H-bond
8	DFG-loop, Asp274: Hydrophobic	H-bond
9	A-loop, His280 : HA	H-bond
10	Lys162 : Ar	Cation- π
11	DFG-loop, Phe169: Ar	π- π
12	A-loop, Trp277 : Ar	π- π
13	A-loop, His280 : Ar	π- π
14	G-loop, Phe144 : Ar	π- π

### 4. Chemotype selectivity of AK binding site pockets

The interactions formed by each individual AK inhibitor in the docked complex with the different binding site residues when studied revealed repetitive participation of select set of residues (S11 Table in S2 File). Therefore, the AK binding site was partitioned into six sub-pockets namely adenine (AP), ribose (RP), phosphate (PP), solvent (SP), back (HPI) and hydrophobic allosteric (HPII) sub-pockets. Each sub-pocket comprises of specific hot-spots and can hold a certain type of pharmacophore. The entire kinase active site provides room for the formation of interactions. However, the pharmacophore features of the known classes of kinase inhibitors allow them to explore only a certain part of the interaction landscape. The comparison of the interacting fragments presents specific chemotypes which show a tendency to bind to the same part of the AK binding site sub-pockets through same set of interactions irrespective of the scaffold it constitutes. The nature of chemotypes differed with the change in conformation indicating the varied inhibitor preferences of different binding-site topologies (Table 4).

**Table 4:** Insights obtained for the design of AK inhibitors.

S.		Inhibitor Class			
No.	Feature	Type I	Type I^1^/_2_	Type II	Type III
1.	Binding	DFG-in,	DFG-in,	DFG-out,	DFG-out,
	Conformation	αC-helix in,	αC-helix in,	αC-helix out,	αC-helix out,
		A-loop in	A-loop in	A-loop out	A-loop out
2.	Binding Pocket	AP, SP	BP, AP, RP, PP	AP, RP, PP, HPII	PP, HPII
3.	Interacting Motifs	Hinge	Hinge,	Hinge,	DFG-loop,
			DFG-loop	DFG-loop,	A-loop,
				αC-helix,	G-loop
				A-loop,	
				G-loop	
4.	Interacting	GK+1, GK+2,	GK, GK+2,	GK+2, GK+3,	A, D, F, W, H, F
	Residues	GK+3, GK+4,	GK+3, GK+4, A,	GK+4, GK+6, K, E,	
		GK+6	D	A, D, F, W, H, F	
5.	Interactions	H-bond	H-bond	H-bond, cation-π,	H-bond, cation-π,
				π-π, CH-π	π-π, CH-π
6.	Hot-spots	2+3	1+2+6	2+3+4+5+6+7	5+6+7
7.	Pharmacophore	HD^1^, HA^2^, HD^2^,	HA^1^, HD^1^, HA^2^,	HD^1^, HA^2^, HD^2^, Ar^1^,	HD^4^, HA^3^, Ar^2^
	features	HD^3^, Ar^1^	HD^2^, Ar^1^, L, HD^4^,	L, HD^4^, HA^3^, Ar^2^	
			HA^3^		
8.	Set	B-C	A⋃B	B⋃C	C

**Figure 5:**
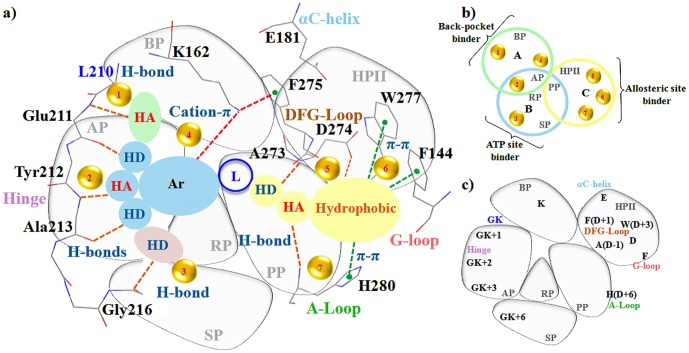
Non-covalent interactions based specificity rendering hot-spots for the design of Aurora kinase inhibitors. The AK binding site has been partitioned into six sub-pockets namely back-pocket (BP), adenine-pocket (AP), sugar-pocket (RP), phosphate-pocket (PP), solvent-pocket (SP) and hydrophobic allosteric-pocket (HPII). All possible pharmacophore features found in different classes of AK inhibitors have been mapped onto these six sub-pockets. The pharmacophore features constitute the H-bond donor (HD), H-bond acceptor (HA), aromatic moiety (Ar), linker (L) and HPII binder (hydrophobic). The Venn diagram shows intersections of the six sub-pockets and hot-spots. The colour of the ring represents the pharmacophore endeared by different sub-pockets. The cartoon representation of the binding-site sub-pockets shows the key interacting residues occupying each sub-pocket. The seven hot-spots highlight the possible non-covalent interactions formed by the key interacting residues of each sub-pocket with different pharmacophore features to achieve specificity.

Flat heterocycles such as quinazolines, dimethyl pyrimidine amine, carboxamino dimethyloxazole of the type I and type II inhibitors bind to the hydrophobic adenine pocket (AP) by forming H-bonds with hinge and gatekeeper (GK). The flat heterocycles comprise of one H-bond acceptor and one or two H-bond donors (Table S11 in File S2). Despite being sequentially conserved, the adenine pocket (AP) derives specificity from the small sized gatekeeper (GK) Leu210 which controls its size. The naphthalene or fluorophenyl of type I ^1^/_2_ scaffolds form H-bond with the back pocket (BP). The adenine pocket (AP) and back pocket (BP) of the sequentially similar kinases such as CDK or PKA differ since they have Phe or Met as gatekeeper (GK). The solvent pocket (SP) of AK consists of several unique residues such as Pro214 (GK+4), Leu215 (GK+5), Thr217 (GK+7) and the αD-helix Arg220 (GK+10) which establish H-bond with H-bond donor to obtain specificity. The ribose pocket (RP) is usually occupied partially by the aromatic rings such as piperidine, nitriles, halogen substituted phenyls and the polar linkers joining the head part of the inhibitor occupying ATP site with the tail part occupying the HPII in type II inhibitors. The H-bond donors of these moieties were seen to form H-bond with the DFG-loop Phe in the inactive conformations. The type I scaffolds occupy the phosphate pocket (PP) to minimum while the linkers such as the urea, methyl acetamide and hydroxyl substituents of the type II scaffolds engage in H-bonds. The hydrophobic allosteric pocket (HPII) is occupied by large chemical entities which constitute of a pair of H-bond donor, acceptor and a hydrophobic moiety. The H-bond donors of the hydrophobic allosteric pocket (HPII) in AK are usually trimethyl groups, nitrogen substituted pyrans, methoxy benzene and aromatic rings with halogens in majority of cases. Based on these analyses, the non-covalent interactions and the complementary specificity rendering sites for the design of different classes of AK inhibitors were prioritized (Fig. 5). The existing inhibitors can be reengineered with sub-pocket specific chemotypes or fragments to achieve specificity [83], [84]. Although the basic framework of kinase inhibitors is identical, the nature of the preferred chemotypes varies.

### 5. Comparative analysis of AK sub-pockets with other kinases

**Figure 6:**
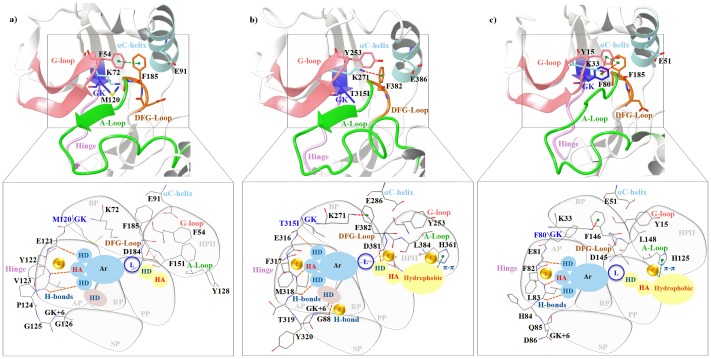
Comparative analysis of the specificity hot-spots explored by AK inhibitor in other kinases. Three kinases a) PKA b) ABL and c) CDK identified as potent co-targets for AK inhibitors have been modelled in the DFG-out (up) conformation. The six AK sub-pockets and its pharmacophore features have been overlapped on the binding-sites of three kinases. The inset shows the influence of the key-residues of different kinases on the binding of AK inhibitors as well as the likely sub-sets of AK specificity rendering sites and non-covalent interactions compatible to other kinases.

The kinases share a high sequence and structural similarity. Therefore, to gauge the propensity of the prioritized hot-spots in rendering specificity to AK, a comparative analysis of AK sub-pockets with other kinases was carried out. Among the kinases with more than 30% sequence identity with AK, the five kinases namely CDK2, JAK2, PKA, ABL1 and ABL2 have been identified as potent co-targets for AK inhibitors (Table 5, Fig. 1). VX-680(MK-0457), AT9283, JNJ-7706621, PHA-739358, YL1-038-31, YL5-083 are the six AK inhibitors which have been co-crystallized with the aforementioned kinases [5], [20], [85]–[88]. The composition of the hinge region residues of AK and PKA vary at Met120 gatekeeper (GK), Gly124 (GK+4), Glu127 (GK+7) and Tyr164 of the HRD motif (AK: Leu210 (GK), Thr215, Gly217, His254) (Fig. 6a). These substitutions influence the topology of the adenine pocket (AP) and therefore the methylpyrazine group of VX-680 protrudes into the SP in PKA unlike AK. The G-loop in PKA is quite distal which makes it inaccessible to the inhibitor. Therefore, VX-680 does not form a π-π interaction with the aromatic Phe54 of the G-loop. Mutation studies by Pflug et al. also validate the role of T183A at D-1, V123A at GK+3, M120L at gatekeeper (GK) in the binding of AK inhibitors VX-680 and JNJ-7706621 to PKA [88]. Likewise they also report that the presence of Leu95 (E+4) in the αC-helix of PKA as against a polar Gln185 (E+4) at the same position in AK influences the H-bond formation with cyclopropane of VX-680 and in turn on the selectivity of back pocket (BP). These factors influence the tight binding of inhibitors and make the inhibitor more specific for AK than PKA. Like PKA, the adenine pocket (AP) of ABL differs from AK due to the presence of a large size residue Phe320 at GK+5 instead of Leu216 as in AK (Fig. 6b). This changes the character of the adenine pocket (AP) and the adjoining solvent pocket (SP). The back pocket (BP) in these two kinases also varies due to the different size of the gatekeeper (GK). While AK has Leu210 as GK, ABL has a Thr315 in the wild-type and Ile315 in mutant. Unlike AK, the G-loop of ABL is in an extended form even after binding of PHA-739358 and the π-π interaction with G-loop is missing. PHA-739358 binds to a DFG-out(up) conformation in AK while in ABL it binds to the DFG-in state. This also highlights the point that a chemotype is capable of inducing a conformational change such as the DFG-flip only if complementary sites are available. This also changes the character of the binding-site sub-pockets. After the T315I mutant of ABL, CDK has been a close attractant of the AK inhibitors. CDK and AK differ in the nature of the adenine pocket (AP) (Fig. 6c). The Gly216 insert in AK makes the adenine pocket (AP) more hydrophobic as compared to CDK. The higher affinity of AT9283 for AK is due to this difference. YL5-083 a bisanilinopyrimidine inhibitor induces the DFG-out(up) conformation by binding to Ala273 (D-1) in AK. While both AK and CDK2 harbour the –ADFG-sequence, the DFG-loop of CDK2 does not adopt the DFG-out(up) conformation even in the presence of induced dipoles created by YL5-083. Despite high structural similarity and considerable sequence similarity shared by AK and CDK2, the N-terminal residues flanking the DFG-loop of CDK2 varies causing a change in the character of the phosphate pocket (PP) and hydrophobic allosteric pocket (HPII). This also suggests that despite the crucial role played by various inhibitor binding mechanisms elucidated hitherto, the design of kinase inhibitors in general and AK in particular depends to a great extent on the formation of interactions with the right sites in target kinase.

**Table 5:** Sequence identity of kinases bound by multi-targeted AK inhibitors with AK.

S. No.	Inhibitor	PDB	UNIPROT identifier	Swiss-prot identifier	Classification (Group/Family/Sub-family)	% Identity*
1.	AT9283	2W1G	O14965	AURKA_HUMAN	Other/Aur	-
		2W1H	P24941	CDK2_HUMAN	CMGC/CDK/CDK2	34
		2W1I	O60674	JAK2_HUMAN	TK/JAK	26
2.	VX-680/MK-0457	3E5A, 4JBQ	O14965	AURKA_HUMAN	Other/Aur	-
		3AMB	P17612	KAPCA_HUMAN	AGC/PKA	33
		2XYN	P42684	ABL2_HUMAN	TK/ABL	27
		2F4J	P00519	ABL1_HUMAN	TK/ABL	27
		4B8M	Q6DE08	AUKBA_XENLA	Other/Aur	70
		4AF3	Q96GD4	AURKB_HUMAN	Other/Aur	74
3.	JNJ-7706621	3AMA	P17612	KAPCA_HUMAN	AGC/PKA	33
4.	PHA-739358	2J50	O14965	AURKA_HUMAN	Other/Aur	-
		2V7A	P00519	ABL1_HUMAN	TK/ABL	27
5.	YL1-038-31	3UO5	O14965	AURKA_HUMAN	Other/Aur	-
		3UNJ	P24941	CDK2_HUMAN	CMGC/CDK/CDK2	34
6.	YL5-083	3UO6	O14965	AURKA_HUMAN	Other/Aur	-
		3UNK	P24941	CDK2_HUMAN	CMGC/CDK/CDK2	34

*A pairwise sequence alignment of AURKA_HUMAN against the individual kinases was done with blast-p to calculate the sequence identity.

## Conclusions

The issue of specificity has been haunting the kinase drug design for decades. The change in the DFG-loop conformation triggers a series of conformational changes which occur in a coordinated fashion. These transitions influence the topology of the active site which is formed by the kinase structural motifs and also the interaction-networks. The major interacting motifs of kinase (αC-helix, G-, DFG- and A-loop) constitute key residues which participate in non-covalent interactions. Non-covalent interactions such as H-Bond, π-π, cation-π and salt-bridge play a major role in stabilizing the kinase conformation through participation in protein-protein and protein-ligand interactions. These interactions participating in inhibitor binding are conformation specific. Thus, their interaction-sites can be used as hot-spots (specificity rendering quotients) for the design of kinase inhibitors.

## Supporting Information

S1 File **S1 Figure**, Test-set used for the evaluation of metric. **S2 Figure**, Metrics for the identification of DFG-loop conformation of kinase based on a) volume of the cone b) sum of four pairwise distances and c) angles. **S3 Figure**, Inter-motif metric based on the centre of mass (COM) for identification of the DFG-loop conformation. The key interacting residues of the major structural motifs participating in conformational-coupling have been identified. The pairwise distance and angles using the COM of their side-chains has been calculated and nine parameters which can most likely be used to distinguish between the DFG-conformations (a) DFG-in, b) DFG-out (up)) of AK have been identified. The nine parameters consist of four distance-based and three angle-based parameters. **S4 Figure**, Contribution and accuracy of the inter-motif metric parameters. a) Contribution of each individual parameter of the inter-motif metric. The crystal structures of AK bound to diverse scaffolds were used to test the performance. Weights (★) have been given to each parameter based on its capacity to distinguish the two DFG-conformations: DFG-in and DFG-out (up). In each graph, the more the distance between the two lines the better is the performance of that parameter. b) Accuracy of the inter-motif parameters in predicting the DFG-loop conformation of Aurora kinase. **S5 Figure**, Intra-motif metric based on centre of mass (COM) for identification of the DFG-loop conformation. The DFG-loop and A-loop residues undergoing maximum variations have been used to identify the nine parameters. The nine parameters consist of four distance-based and three angle-based parameters whose pairwise distance and angles have been used as a measure to distinguish the DFG-conformation (a) DFG-in, b) DFG-out (up)) of AK. **S6 Figure**, Contribution and accuracy of the intra-motif metric parameters. a) Contribution of each individual parameter of the intra-motif metric. The crystal structures of AK bound to diverse scaffolds were used to test the performance. Weights (★) have been given to each parameter based on its capacity to distinguish the two DFG-conformations: DFG-in and DFG-out (up). In each graph, the more the distance between the two lines the better is the performance of that parameter. b) Accuracy of the intra-motif parameters in predicting the DFG-conformation of AK. **S7 Figure**, Kinase signature profile of AK generated from Kinase Sequence Database. The profile shows points in the AK sequence which contains unique (non-conserved) residues. The height of the bar is proportional to the uniqueness of that residue. Red bars correspond to ≥95% uniqueness which means that the residue at that particular position is found in ≤5% of kinases. Orange bars correspond to residues found in 5-10% sequences and yellow bars correspond to those between 10-15%. If at a given position there are more than 50% insertions (-) then the corresponding bar is coloured grey. The binding site contact residues are highlighted in green and the gatekeeper in red. **S8 Figure**, Impact of conformational transitions on the major structural motifs (a-c) of the four studied conformations. **S9 Figure**, The conformational variations in the DFG-loop, αC-helix and G-loop of AK in the 40 ns molecular dynamics simulation. The differences have been measured by calculating the back-bone RMSD of these major structural motifs.
(DOCX)

S2 File **S1 Table**, Analysis of the crystal structures of AK of all organisms from Protein Data Bank (PDB). **S2 Table**, Sorting of AK structures and co-crystals from Protein Data Bank (PDB) according to sequence type and position. **S3 Table**, Identification of kinases sequentially similar to AK through pairwise sequence alignment of AURKA_HUMAN against the entire kinome present in kinbase v1.1 using blast-p. **S4 Table**, Geometric parameters of the inter-residue metric for the identification of DFG-loop conformation in kinase based on centre of mass (COM). **S5 Table**, Performance of the inter-residue metric based on centre of mass (COM) in identifying the DFG-loop conformation of AK. **S6 Table**, Prioritizing the parameters of the inter-motif metric based on their performance in distinguishing the DFG-conformation of AK. **S7 Table**, Geometric parameters of the intra-motif DGF- and A-loop metric for the identification of DFG-loop conformation in kinase based on centre of mass (COM). **S8 Table**, Performance of the intra-residue DFG- and A-loop metric based on centre of mass (COM) in identifying the DFG-loop conformation of AK. **S9 Table**, Prioritizing the parameters of the intra DFG- and A-loop motif metric based on their performance in distinguishing the DFG conformation of AK. **S11 Table**, Interacting chemotypes of AK co-crystals present in Protein Data Bank (PDB).
(DOC)

S3 File **S10 Table**, a) Scoring component of the lowest RMSD pose of Aurora kinase co-crystal complexes obrained through Glide 4.5 docking. b) Score and RMSD of the best scoring and lowest RMSD poses obtained on docking Aurora kinase with it's co-crystals' using Glide 4.5.
(XLSX)
