# Supplementary material for: Specificity Rendering ‘Hot-Spots’ for Aurora Kinase Inhibitor Design: The Role of Non-Covalent Interactions and Conformational Transitions
Source: PLoS One. 2014 Dec 8;9(12):e113773. doi: 10.1371/journal.pone.0113773 (PMC4259475; doi:10.1371/journal.pone.0113773)
Supplement: S1 File — S1 Figure, Test-set used for the evaluation of metric. S2 Figure, Metrics for the identification of DFG-loop conformation of kinase based on a) volume of the cone b) sum of four pairwise distances and c) angles. S3 Figure, Inter-motif metric based on the centre of mass (COM) for identification of the DFG-loop conformation. The key interacting residues of the major structural motifs participating in conformational-coupling have been identified. The pairwise distance and angles using the COM of their side-chains has been calculated and nine parameters which can most likely be used to distinguish between the DFG-conformations (a) DFG-in, b) DFG-out (up)) of AK have been identified. The nine parameters consist of four distance-based and three angle-based parameters. S4 Figure, Contribution and accuracy of the inter-motif metric parameters. a) Contribution of each individual parameter of the inter-motif metric. The crystal structures of AK bound to diverse scaffolds were used to test the performance. Weights (★) have been given to each parameter based on its capacity to distinguish the two DFG-conformations: DFG-in and DFG-out (up). In each graph, the more the distance between the two lines the better is the performance of that parameter. b) Accuracy of the inter-motif parameters in predicting the DFG-loop conformation of Aurora kinase. S5 Figure, Intra-motif metric based on centre of mass (COM) for identification of the DFG-loop conformation. The DFG-loop and A-loop residues undergoing maximum variations have been used to identify the nine parameters. The nine parameters consist of four distance-based and three angle-based parameters whose pairwise distance and angles have been used as a measure to distinguish the DFG-conformation (a) DFG-in, b) DFG-out (up)) of AK. S6 Figure, Contribution and accuracy of the intra-motif metric parameters. a) Contribution of each individual parameter of the intra-motif metric. The crystal structures of AK bound to diverse scaffolds were [file pone.0113773.s001.docx]

**Figure S1.** Test-set used for the evaluation of metric.

| **a)**  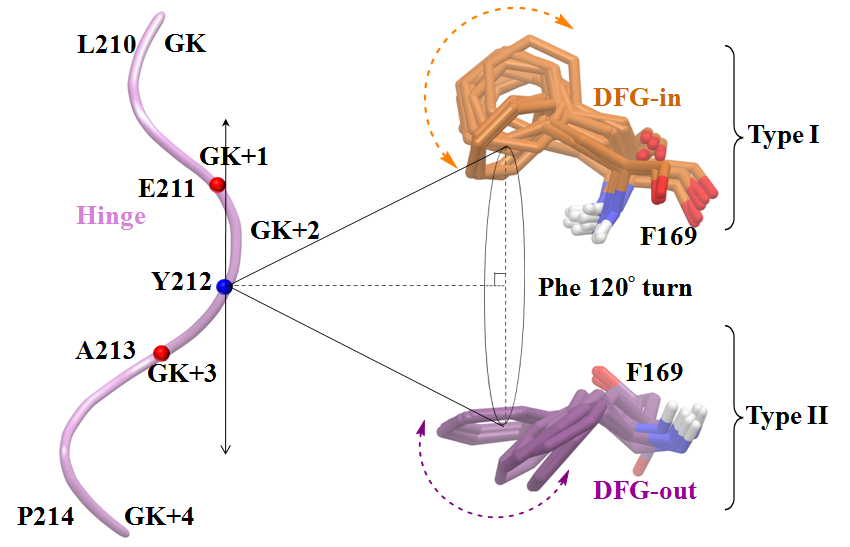 | ${\text{A = }\frac{\text{1}}{\text{2}}\text{V}}_{\text{DFG-in}}$ Eq. (1)  ${\text{B = }\frac{\text{1}}{\text{2}}\text{V}}_{\text{DFG-out}}$ Eq. (2)  $\text{V=}\frac{\text{1}}{\text{3}}\text{πr}^{\text{2}}\text{h}$ =$\text{ A + B}$ Eq. (3)  $\text{∴}\text{ If }\frac{\text{1}}{\text{2}}\text{V}_{\text{unknown}}\text{ = A, }\text{C}_{\text{unknown}}\text{ = DFG-in}$ Eq. (4)  $\text{∴}\text{ If }\frac{\text{1}}{\text{2}}\text{V}_{\text{unknown}}\text{= B, }\text{C}_{\text{unknown}}\text{ = DFG-out}$ Eq. (5) |
| --- | --- |
| where, V = Volume of the cone comprising the vertices of hinge GK+2 residue and the centres of the conformational space occupied by the DFG-loop in the ′in′ and ′out′ conformations. ′A ′ and ′ B ′ = Average of the DFG-loops of all known structures used as a threshold. Cunknown = Conformation of unknown based on the range in which ½ of the ′ V ′ falls in. | |
| **b)**  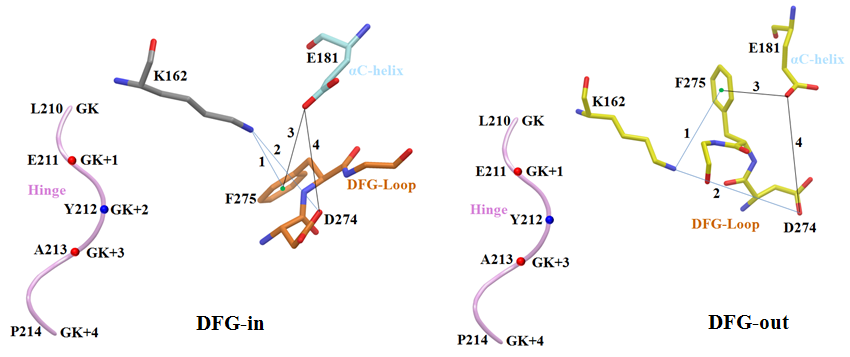  If (1+2+3+4)≤30Å,coformation=D_(DFG-in) Eq. (6)  If (1+2+3+4) ≥32Å,coformation=D_(DFG-out) Eq. (7)  If (1+2+3+4) =(30-32Å),coformation=D_(pseudo DFG-out) Eq. (8) | |
| where, 1= Distance between Lys162 (NZ) – Phe275 (centroid); 2= Distance between Lys162(NZ) – Asp274 (OD1); 3= Distance between Glu181(OE1) – Phe275 (centroid); 4= Distance between Glu181(OE1) – Asp274 (OD1) | |
| **c)**  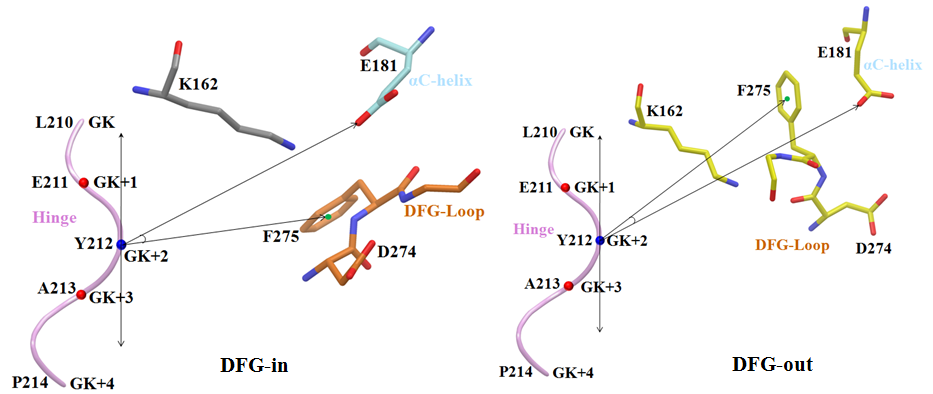 | |

**Figure S2.** Metrics for the identification of DFG-loop conformation of kinase based on a) volume of the cone b) sum of four pairwise distances and c) angles.


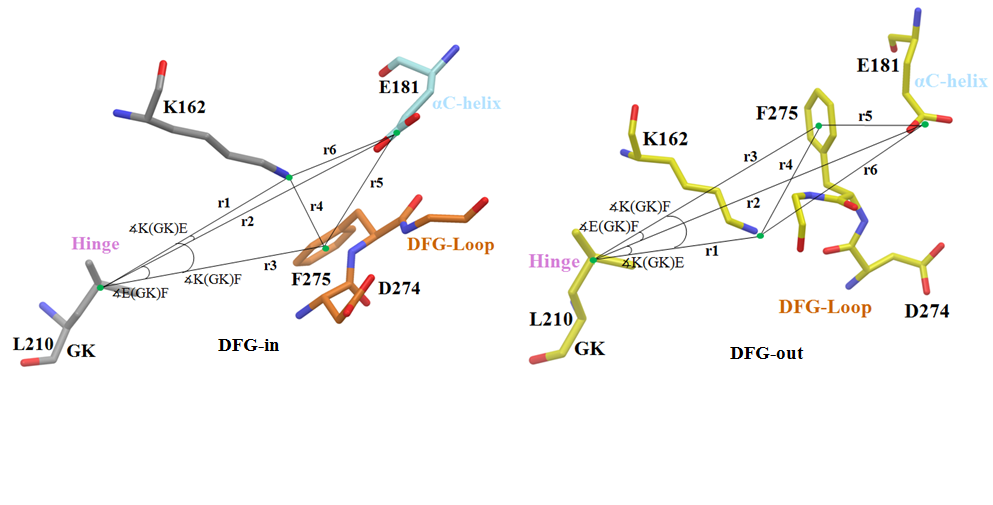


**Figure S3. Inter-motif metric based on the centre of mass (COM) for identification of the DFG-loop conformation.** The key interacting residues of the major structural motifs participating in conformational-coupling have been identified. The pairwise distance and angles using the COM of their side-chains has been calculated and nine parameters which can most likely be used to distinguish between the DFG-conformations (a) DFG-in, b) DFG-out (up)) of AK have been identified. The nine parameters consist of four distance-based and three angle-based parameters.

| **a)**  **** | |
| --- | --- |
| **b)**  **** | *Legend:  P1: r1(GK …K162),  P2: r2(GK …E181),  P3: r3(GK …F275),  P4: r4(K162 …F275),  P5: r5(E181 …F275),  P6: r6(K162 …E181),  P7: ∡1 (K...GK...E),  P8: ∡2 (K...GK...F),  P9: ∡3 ([E...GK...F) |

**Figure S4.** **Contribution and accuracy of the inter-motif metric parameters.** a) Contribution of each individual parameter of the inter-motif metric. The crystal structures of AK bound to diverse scaffolds were used to test the performance. Weights (★) have been given to each parameter based on its capacity to distinguish the two DFG-conformations: DFG-in and DFG-out (up). In each graph, the more the distance between the two lines the better is the performance of that parameter. b) Accuracy of the inter-motif parameters in predicting the DFG-loop conformation of Aurora kinase.

**Figure S5.** **Intra-motif metric based on centre of mass (COM) for identification of the DFG-loop conformation.** The DFG-loop and A-loop residues undergoing maximum variations have been used to identify the nine parameters. The nine parameters consist of four distance-based and three angle-based parameters whose pairwise distance and angles have been used as a measure to distinguish the DFG-conformation (a) DFG-in, b) DFG-out (up)) of AK.

| **a)**  **** | |
| --- | --- |
| **b)**  **** | *Legend:  P1: r1 (A273...D274)  r2 (D274...F275)  r3 (F275-G276)  r4 (D274...G276)  r5 (F275-W277)  r6 (F275...T288)  ∡1 (DFG)  ∡2 (FWH)  ∡3 (FWT) |

**Figure S6.** **Contribution and accuracy of the intra-motif metric parameters.** a) Contribution of each individual parameter of the intra-motif metric. The crystal structures of AK bound to diverse scaffolds were used to test the performance. Weights (★) have been given to each parameter based on its capacity to distinguish the two DFG-conformations: DFG-in and DFG-out (up). In each graph, the more the distance between the two lines the better is the performance of that parameter. b) Accuracy of the intra-motif parameters in predicting the DFG-conformation of AK.


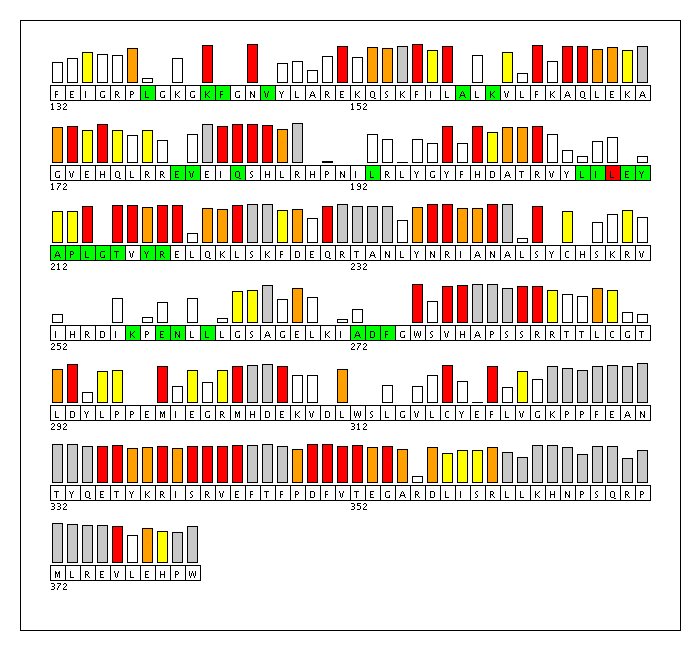


**Figure S7. Kinase signature profile of AK** **generated from Kinase Sequence Database.** The profile shows points in the AK sequence which contains unique (non-conserved) residues. The height of the bar is proportional to the uniqueness of that residue. Red bars correspond to ≥ 95 % uniqueness which means that the residue at that particular position is found in ≤ 5 % of kinases. Orange bars correspond to residues found in 5-10% sequences and yellow bars correspond to those between 10-15%. If at a given position there are more than 50% insertions (-) then the corresponding bar is coloured grey. The binding site contact residues are highlighted in green and the gatekeeper in red.

| **a) 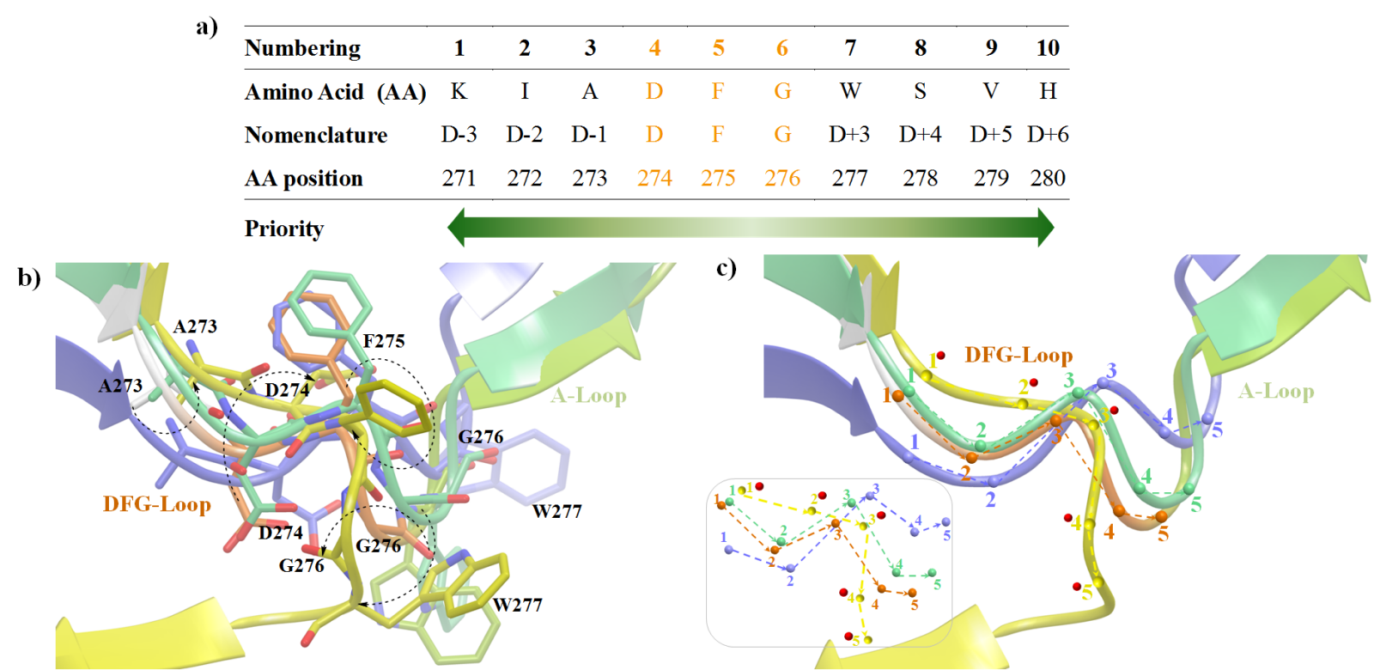** | **DFG-loop and A-loop conformational transitions** a) The DFG-loop sequence along with the residues of the catalytic and A-loop flanking it. The residues have been named with respect to the position of DFG-loop Asp274. b) Backbone-overlap of the DFG-loop and A-loop (2W1C: DFG-in, aquamarine; 3UNZ: DFG-out (up), yellow; 3E5A: DFG-in, pink; 3DJ6: DFG-in, blue) of AK highlighting the structural variations occurring on the DFG-loop flip. c) Positional variations in the residues of the DFG-loop and A-loop as a result of conformational transition. |
| --- | --- |
| **b)**  **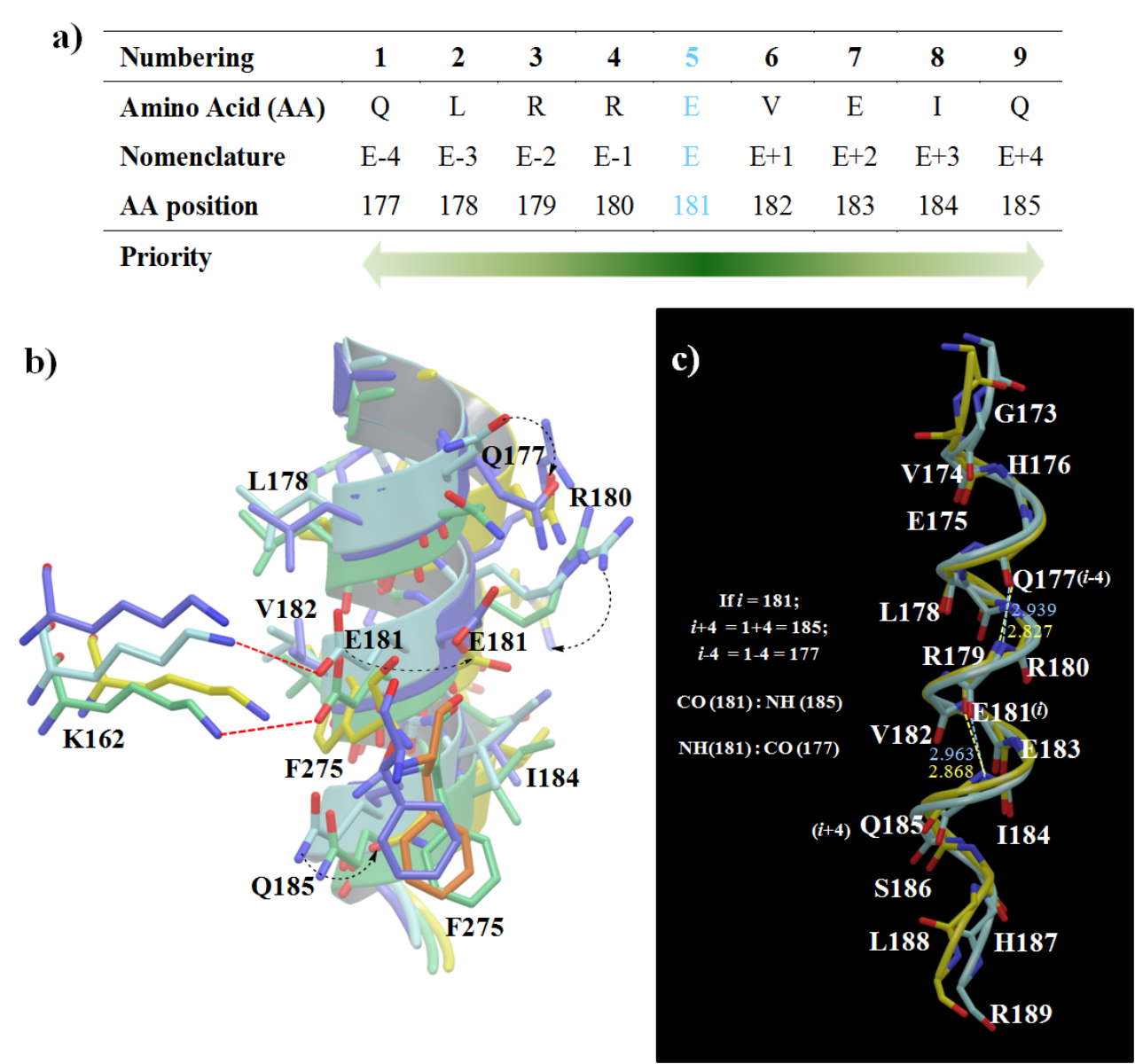** | **αC-helix conformational transitions** a) Amino acid residues forming the αC-helix of AK. The residues have been named with respect to the position of the salt-bridge former E181. b) Backbone-overlap of the αC-helix of AK highlighting residues flanking E181 of the four studied conformations (2W1C: αC-helix in, aquamarine; 3UNZ: αC-helix out, yellow; 3E5A: αC-helix in, pink; 3DJ6: αC-helix out, blue). b) Impact of the αC-helix rotation occurring in response to the DFG-loop transition on the nature of H-bonding required for the stabilization of the right-handed αC-helix. |
| **c)**  **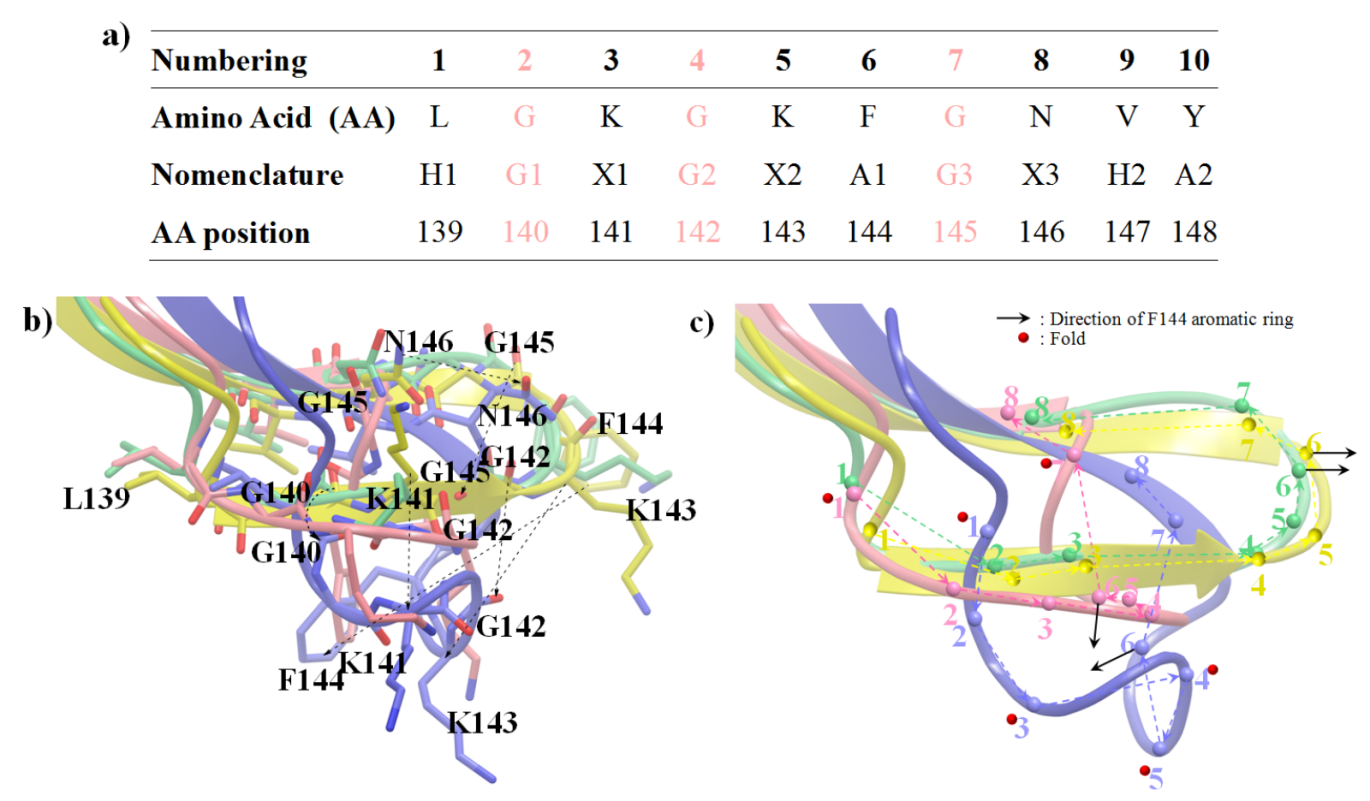** | **G-loop conformational transitions** a) Amino acid residues constituting the G-loop and its flanking regions in Aurora kinase. b) Backbone-overlap of the G-loop conformations (2W1C: G-loop extended, aquamarine; 3UNZ: G-loop extended, yellow; 3E5A: G-loop folded, pink; 3DJ6: G-loop folded, blue) highlighting the position of the -XGXGXXGXX- motif. The arrows indicate the conformational variation of each of the G-loop amino acid residue in the extended and folded conformations. **c**) Comparative arrangement of the G-loop residues and the positional variation of specific residues leading to a fold in the G-loop. |

**Figure S8.** Impact of conformational transitions on the major structural motifs (a-c) of the four studied conformations.

| a) System-I:2W1C (D_I_, C_I_, G_E_ )   | d) System-IV: 3UNZ (D_OU_, C_O_, G_F_ )   |
| --- | --- |
| b) System-II:3E5A (D_I_, C_I_, G_F_ )   | e) System-V:apo (D_I_, C_I_, G_E_ )   |
| c) System-III:3DJ6 (D_I_, C_O_, G_F_ )   | f) System-VI: apo (D_OU_, C_O_, G_E_ )   |
|  | |

**Figure S9. The conformational variations in the DFG-loop, αC-helix and G-loop of AK in the 40 ns molecular dynamics simulation.** The differences have been measured by calculating the back-bone RMSD of these major structural motifs.
