# Supplementary material for: Specificity Rendering ‘Hot-Spots’ for Aurora Kinase Inhibitor Design: The Role of Non-Covalent Interactions and Conformational Transitions
Source: PLoS One. 2014 Dec 8;9(12):e113773. doi: 10.1371/journal.pone.0113773 (PMC4259475; doi:10.1371/journal.pone.0113773)
Supplement: S2 File — S1 Table, Analysis of the crystal structures of AK of all organisms from Protein Data Bank (PDB). S2 Table, Sorting of AK structures and co-crystals from Protein Data Bank (PDB) according to sequence type and position. S3 Table, Identification of kinases sequentially similar to AK through pairwise sequence alignment of AURKA_HUMAN against the entire kinome present in kinbase v1.1 using blast-p. S4 Table, Geometric parameters of the inter-residue metric for the identification of DFG-loop conformation in kinase based on centre of mass (COM). S5 Table, Performance of the inter-residue metric based on centre of mass (COM) in identifying the DFG-loop conformation of AK. S6 Table, Prioritizing the parameters of the inter-motif metric based on their performance in distinguishing the DFG-conformation of AK. S7 Table, Geometric parameters of the intra-motif DGF- and A-loop metric for the identification of DFG-loop conformation in kinase based on centre of mass (COM). S8 Table, Performance of the intra-residue DFG- and A-loop metric based on centre of mass (COM) in identifying the DFG-loop conformation of AK. S9 Table, Prioritizing the parameters of the intra DFG- and A-loop motif metric based on their performance in distinguishing the DFG conformation of AK. S11 Table, Interacting chemotypes of AK co-crystals present in Protein Data Bank (PDB). (DOC) [file pone.0113773.s002.doc]

**Table S1.** Analysis of the crystal structures of AK of all organisms from Protein Data Bank (PDB).

| **Sr.**  **No.** | **PDB**  **id** | **Position** | | | **Breaks** | **MODRES** | **Side Chain refinement** |
| --- | --- | --- | --- | --- | --- | --- | --- |
|  |  | **Sequence** | **Structure** | |  |  |  |
| ***Homo sapiens*** | | | | | | | |
|  | [**1MQ4**](http://www.rcsb.org/pdb/explore.do?structureId=1MQ4) | 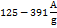 | R126-S388 | R285-T288 | | - | R126, Q127, E170, K171, E175, E183, K339, R375 |
|  | [**1MUO**](http://www.rcsb.org/pdb/explore.do?structureId=1MUO) | 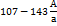 | W128-S388 | S278-L289 | | - | E134, K141, K143, K153, Q154, K156, L169, E170, V174, E175, H176, R180, K309 |
|  | [**1OL5**](http://www.rcsb.org/pdb/explore.do?structureId=1OL5) | 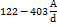 | S123-S388 | - | | T - TPO287;  T - TPO288 | R375 |
|  |  | 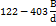 | S7-N43 | S21-N30 | | - | E36 |
|  | **1OL6** | 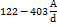 | W128-S387 | K141-K143;  A172-E175;  H280-T292;  G303-M305 | | - | E131, K141, K143, N146, R151, K153, Q154, K156, F165, Q168, E170, K171, H176, Q177, R179, R180, E183, R189, H201, D202, T204, Y207, R220, K227, Q231, R251, L293, E302, M305, D307, L326, M332, Y334, Q335, T337, Y338, K339, R340, R343, E354, R375 |
|  | **1OL7** | 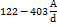 | Q127-K389 | - | | T - TPO287;  T - TPO288 | K171, R220, K250, R285, L289, C290, M305, E336, K339, E354, R375, K389 |
|  | **2BMC** | 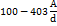 | Q127-K389 | - | | - | - |
|  |  | 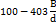 | K125-V279 | - | | - | - |
|  |  | 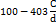 | K125-V279 | - | | - | - |
|  |  | 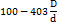 | Q127-K389 | - | | - | - |
|  |  | 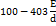 | Q127-K389 | - | | - | - |
|  |  | 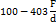 | R126-V279 | - | | - | - |
|  | **2C6D** | 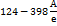 | R125-K388 | H279-G290 | | - | R125, E130, K140, K142, L177, R179, E182, H279, R374 |
|  | **2C6E** | 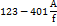 | Q126-K388 | S277-G290 | | - | E130, K140, K142, R188, T203, R374 |
|  |  | 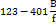 | Q126-S387 | D273-G290 | | - | E130, K140, K142, R188, T203, R374, R219, R250, I252, E354 |
|  | **2DWB** | 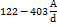 | R126-K389 | R285-T288 | | M – MSE300;  M – MSE305;  M – MSE373 | - |
|  | **2J4Z** | 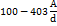 | R126-S388 | - | | - | - |
|  |  | 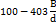 | R126-Q394 | - | | - | - |
|  | **2J50** | 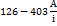 | W128-S388 | V279-T288;  E302-D307 | | - | V279 |
|  |  | 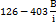 | W128-S388 |  |  | - |  |
|  | **2NPQ** | 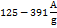 | Q127-S388 | R285-T292 | | - | - |
|  | **2W1C** | 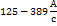 | Q127-K389 | - | | T - TPO287;  T - TPO288 | L289, C290, E336 |
|  | **2W1D** | 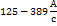 | Q127-K389 | V279-G291;  D307-K309 | | - | E336 |
|  | **2W1E** | 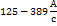 | Q127-K389 | V279-G291 | | - | E336 |
|  | **2W1F** | 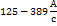 | Q127-K389 | V279-G291 | | - | E336 |
|  | **2W1G** | 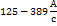 | Q127-K389 | A281-G291 | | - | E336 |
|  | **2WQE** | 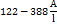 | Q127-S388 | D274-G291;  I301-D307 | | - | Q127, K141, K143, V174, H176, Q177, R180, E181, E183, Q231, R251, R255, D307, Y334, E354 |
|  | **2WTV** | 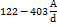 | K125-K389 | - | | T - TPO287;  T - TPO288;  C – CME290 | K156, A281, K326, R375 |
|  |  | 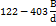 | R126-K389 | - | |  | R126, Q127, R251, K339, E354, R375 |
|  |  | 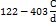 | R126-K389 | - | |  | R126, Q127, R189, E217, Q335, K339, E375 |
|  |  | 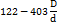 | K125-K389 | - | |  | K141, R179, K326, K339, R375 |
|  | **2WTW** | 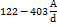 | W128-K389 | S283-C290 | | - | Q169, L169, K171, R179, K250, R251, E307 |
|  | **2X6D** | 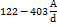 | R126-S388 | E170-E175;  R285-C290 | | - | R126, Q127, E152, E183, T217, R220, H280, R285, K339, R375 |
|  | **2X6E** | 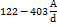 | R126-S388 | H280-T292;  E302-H306 | | - | R126, Q127, K153, K156, K166, E170, K171, E175, R180, E183, R189, K224, R251, K339, E354, R362, L374, R375, E379 |
|  | **2X81** | 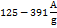 | Q127-S388 | G140-F144;  A273-G291 | | - | Q127, F144, Q154, K166, E170, K171, E175, H176, Q177, E179, R180, E181, E183, I184, Q185, H187, D202, R220, K224, K250, R251,  M305, E336, K339, E354, R375 |
|  | **2XNE** | 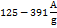 | Q127-S388 | Q127-G173;  P282-T292 | | - | K141, K143, F144, Q154, K156, E175, H176, Q177, R179, R180, E183, I184, R189, R195, R220, K250, R251, M305, E308, E336, K339, E334, R375 |
|  | **2XNG** | 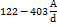 | Q127-K389 | A281-T292 | | - | Q127, E131, K141, K143, M146, K166, Q168, L169, E170, K171, V174, E175, H176, Q177, R179, R180, E183, R189, R205, R220, K224, Q231, K250, R251, W277, H280, M305, E336, K339, E354, R375, K389 |
|  | **2XRU** | 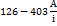 | R126-S388 | V279-C290 | | - | - |
|  | **3COH** | 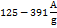 | W128-S388 | D274-L289 | | - | - |
|  |  | 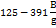 |  |  |  | - | - |
|  | **3E5A** | 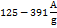 | K125-K389 | - | | T – TPO288 | - |
|  |  | 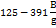 | S6-E42 | S21-G26 | | - | S21, D27, T28, K389 |
|  | **3EFW** | 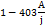 | R126-S388 | F275-L293;  E302-K309 | | - | R126, K143, R151, K156, Q177, R179, R180, E181, H248, K250, R251, R255, K255, M332, Y334, Q335 |
|  |  | 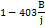 | R126-K389 | F275-L293;  E302-E308 | | - | Q127, K143, K166, E170, K171, R179, R180, R251, R255, Y334, Q335 |
|  | **3FDN** | 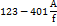 | R126-Q394 | A281-C290 | | - | R126, Q394 |
|  | **3HOY** | 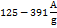 | R126-S388 | S278-T292 | | - | - |
|  | **3HOZ** | 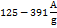 | W128-S388 | S278-L293 | | - | - |
|  |  | 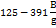 | R126-S388 | G140-V147,  D274-T292 | | - | - |
|  |  | 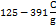 | R126-S388 | D274-T292 | | - | - |
|  | **3H10** | 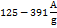 | A124-S391 | - | | - | - |
|  |  | 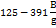 | K125-S391 | - | | - | - |
|  |  | 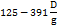 | R126-S388 | S283-T292 | | - | - |
|  | **3HA6** | 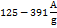 | G124-S388 | - | | - | - |
|  |  | 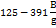 | M1-E42 | L22-N30 | | T - TPO287;  T - TPO288 | S21, L22, N30, E42 |
|  | **3K5U** | 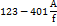 | R126-Q394 | H280-T292 | | - | R126 |
|  | **3LAU** | 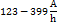 | M124-S391 | F275-L293 | | - | - |
|  | **3MYG** | 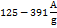 | R126-S388 | H280-T292 | | - | - |
|  | **3NRM** | 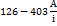 | R126-S388 | - | | - | - |
|  | **3O50** | 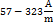 | R126-S388 | W277-T292;  E302-H306 | | - | R126, K143, Q177, K250, R251, R255, W277, N332, Y334, Q335 |
|  |  | 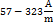 | R126-K389 | F275-T287;  E302-D307 | | - | Q127, K143, L178, R180, R251, I253, T287,Y334, Q335 |
|  | **3O51** | 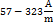 | R126-S388 | F275-L293 | | - | R126, K162, R179, R180, K250, R251, R255, R304, M305, Y334, Q335 |
|  |  |  |  |  | | ***Xenopus laevis*** |  |
|  | **2BFX** | 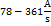 | R86-Y355 | - | | - | - |
|  |  | 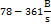 | T78-Q356 | - | | - | - |
|  |  | 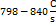 | I798-P837 | - | | - | - |
|  |  | 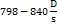 | I798-S840 | - | | - | - |
|  | **2BFY** | 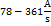 | R86-Q356 | - | | T - TPO248 | - |
|  |  | 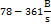 | T78-Q358 | - | | T - TPO248 | - |
|  |  | 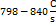 | A800-F837 | - | | - | S803 |
|  |  | 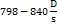 | N805-F837 | - | | - | - |
|  | **2VGO** | 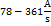 | K87-Y355 | - | | T - TPO248 | E177, L272 |
|  |  | 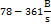 | T78-S357 | K131-V134 | | T - TPO248 | S291, S321 |
|  |  | 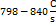 | P797-F837 | - | | - | S803 |
|  |  | 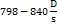 | I798-S840 | - | | - | - |
|  | **2VGP** | 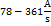 | F88-Q356 | - | | T - TPO248 | - |
|  |  | 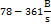 | T78-Y355 | - | | T - TPO248 | - |
|  |  | 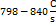 | I798-F837 | G102-N106 | | - | R823 |
|  |  | 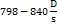 | I798-S840 | - | | - | - |
|  | **2VRX** | 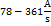 | K87-Y355 | - | | T - TPO248 | D91, S146,, S291, R325 |
|  |  | 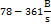 | N77-Y355 | G100-G105 | | T - TPO248 | S321 |
|  |  | 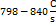 | I798-F837 | - | | - | - |
|  |  | 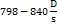 | I798-S840 | - | | - | - |
|  |  |  |  |  | | ***Mus musculus*** |  |
|  | **3D14** | 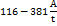 | G113-S401 | S137-R139;  S296-C303 | | - | Q140, K156, K184, R193, K263 |
|  | **3D15** | 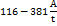 | R139-S401 | S296-G304 | | - | Q140, R192, R233 |
|  | **3D2I** | 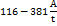 | Q140-S401 | P295-C303 | | - | Q140, K154, K156, K179, K184, E188, R193, E196, K263, R264 |
|  | **3D2K** | 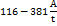 | T141-S401 | H293-T305;  I314-D320 | | - | K154, K184, R192, R193, R268 |
|  | **3DAJ** | 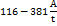 | Q127-S388 | S278-T292;  I301H306 | | - | Q127, K141, K171, E175, R180, E183, K250 |
|  | **3DJ5** | 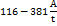 | W141-S401 | W290-T300 | | - | - |
|  | **3DJ6** | 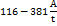 | W141-S401 | W290-T300 | | - | - |
|  | **3DJ7** | 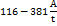 | R139-S401 | S296-C303 | | - | R139, Q140, K156, Q181, E183, K184, R192, K193, R218, K237, K263, K322, E349 |

| R126-S388 | W128-S388 | Q127-K389 | K125-V279 | R126-Q394 | Q127-S388 |
| --- | --- | --- | --- | --- | --- |
| K125-K389 | R126-K389 | K87-Y355 | I798-F837 | R139-S401 | W141-S401 |

**Table S2. Sorting of AK structures and co-crystals from Protein Data Bank (PDB) according to sequence type and position.**

| **UNIPROT** | **Position** | **Chain type** | **PDB id** | **No. of Structures** | **No. of Co-crystals** |
| --- | --- | --- | --- | --- | --- |
| ***Homo sapiens*** | | | | |  |
| **O14965** | 107-403 | A | 1MUO_ADN | 1 | 1 |
|  | 122-388 | A | 2WQE_ADP | 1 | 1 |
|  | 122-389 | A | 2W1C_L0C, 2W1C_TPO, 2W1D_L0D2, W1E_L0E, 2W1F_L0F, 2W1G_L0G, 2W1G_TPO | 5 | 7 |
|  | 122-403 | A | 2WTW_ZZL, 2X6D_X6D, 2X6D_SO4, 1OL5_ADP_A, 1OL5_MG_A, 1OL5_SO4_A, 1OL5_TPO_A, 1OL6_ATP, 1OL7_ADP, 1OL7_MG, 1OL7_TPO, 2DWB_ANP, 2DWB_MSE, 2DWB_SO4, 2X6E_YM4, 2XNG_A0H | 8 | 16 |
|  |  | A/B/C/D | 2WTV_ACT_A, 2WTV_CME_A, 2WTV_DMS_A, 2WTV_EDO_A, 2WTV_TPO_A, 2WTV_ZZL_A, 2WTV_ACT_B, 2WTV_CME_B, 2WTV_DMS_B, 2WTV_EDO_B, 2WTV_TPO_B, 2WTV_ZZL_B, 2WTV_ACT_C, 2WTV_CME_C, 2WTV_EDO_C, 2WTV_TPO_C, 2WTV_ZZL_C, 2WTV_ACT_D, 2WTV_CME_D, 2WTV_EDO_D, 2WTV_TPO_D, 2WTV_ZZL_D | 1 | 22 |
|  | 124-398 | A | 2C6D_ANP, 2C6D_GOL, 2C6D_PO4 | 1 | 3 |
|  | 123-401 | A | 3FDN_MMH, 3K5U_PFQ_A, 3K5U_PFQ_B | 2 | 3 |
|  |  | A/B | 2C6E_HPM_A, 2C6E_HPM_B | 1 | 2 |
|  | 125-391 | A | 2X81_ZZL, 1MQ4_ADP, 1MQ4_MG, 1MQ4_PO4, 2NP8_CC3, 2NP8_SO4, 2XNE_ASH, 3E5A_SO4_A, 3E5A_TPO_A, 3E5A_VX6_A, 3H0Y_48B_A, 3H0Y_SO4_A, 3H0Y_48B_B, 3H0Y_SO4_B, 3HA6_2JZ_A, 3HA6_TPO_A, 3MYG_EML, 3MYG_PG4, 3O51_LJF, 3E5A_SO4_A, 3E5A_TPO_A, 3E5A_VX6_A, 3HA6_2JZ_A, 3HA6_TPO_A | 11 | 24 |
|  |  | A/B | 3COH_83H_A, 3COH_83H_B, 3O50_LJE_A, 3O50_LJE_B, | 4 | 4 |
|  |  | A/B/C | 3H0Z_45B_A, 3H0Z_45B_B, 3H0Z_45B_C | 2 | 3 |
|  |  | A/B/D | 3H10_97B_A, 3H10_97B_B, 3H10_97B_D | 1 | 3 |
|  | 125-399 | A | 3LAU_OFI | 1 | 1 |
|  | 126-403 | A | 2XRU_400, 3NRM_NRM, 1OL5_ADP_A, 1OL5_MG_A | 3 | 4 |
|  |  | A/B | 2J50_627_A, 2J50_SO4_A, 2J50_627_B, 2J50_SO4_B | 1 | 4 |
|  | 1-403 | A/B | 3EFW_AK8_A, 3EFW_SO4_A, 3EFW_AK8_B, 3EFW_SO4_B | 1 | 4 |
|  | 100-403 | A/B | 2J4Z_626_A, 2J4Z_ARS_A, 2J4Z_626_B, 2J4Z_ARS_B | 1 | 4 |
|  |  | A/B/C/D | 2BMC_MPY_A, 2BMC_MPY_B, 2BMC_MPY_C, 2BMC_MPY_D, 2BMC_MPY_E, 2BMC_MPY_F | 1 | 6 |
| ***Xenopus Laevis*** | | | | | |
| **O13024** | 798-840 ^q^ | C/D | [2BFX](http://www.rcsb.org/pdb/explore.do?structureId=2BFX)_[TPO](http://www.rcsb.org/pdb/ligand/ligandsummary.do?hetId=TPO)_C, [2BFX](http://www.rcsb.org/pdb/explore.do?structureId=2BFX)_[TPO](http://www.rcsb.org/pdb/ligand/ligandsummary.do?hetId=TPO)_D, [2BFY](http://www.rcsb.org/pdb/explore.do?structureId=2BFY)_[H1N](http://www.rcsb.org/pdb/ligand/ligandsummary.do?hetId=H1N)_C, [2BFY](http://www.rcsb.org/pdb/explore.do?structureId=2BFY)_[TPO](http://www.rcsb.org/pdb/ligand/ligandsummary.do?hetId=TPO)_C, [2BFY](http://www.rcsb.org/pdb/explore.do?structureId=2BFY)_[H1N](http://www.rcsb.org/pdb/ligand/ligandsummary.do?hetId=H1N)_D, [2BFY](http://www.rcsb.org/pdb/explore.do?structureId=2BFY)_[TPO](http://www.rcsb.org/pdb/ligand/ligandsummary.do?hetId=TPO)_D, [2VGO](http://www.rcsb.org/pdb/explore.do?structureId=2VGO)_[AD5](http://www.rcsb.org/pdb/ligand/ligandsummary.do?hetId=AD5)_C, [2VGO](http://www.rcsb.org/pdb/explore.do?structureId=2VGO)_[TPO](http://www.rcsb.org/pdb/ligand/ligandsummary.do?hetId=TPO)_C, [2VGO](http://www.rcsb.org/pdb/explore.do?structureId=2VGO)_[AD5](http://www.rcsb.org/pdb/ligand/ligandsummary.do?hetId=AD5)_D, [2VGO](http://www.rcsb.org/pdb/explore.do?structureId=2VGO)_[TPO](http://www.rcsb.org/pdb/ligand/ligandsummary.do?hetId=TPO)_D, [2VGP](http://www.rcsb.org/pdb/explore.do?structureId=2VGP)_[AD6](http://www.rcsb.org/pdb/ligand/ligandsummary.do?hetId=AD6)_C, [2VGP](http://www.rcsb.org/pdb/explore.do?structureId=2VGP)_[TPO](http://www.rcsb.org/pdb/ligand/ligandsummary.do?hetId=TPO)_C, [2VGP](http://www.rcsb.org/pdb/explore.do?structureId=2VGP)_[AD6](http://www.rcsb.org/pdb/ligand/ligandsummary.do?hetId=AD6)_D, [2VGP](http://www.rcsb.org/pdb/explore.do?structureId=2VGP)_[TPO](http://www.rcsb.org/pdb/ligand/ligandsummary.do?hetId=TPO)_D, [2VRX](http://www.rcsb.org/pdb/explore.do?structureId=2VRX)_[447](http://www.rcsb.org/pdb/ligand/ligandsummary.do?hetId=447)_C, [2VRX](http://www.rcsb.org/pdb/explore.do?structureId=2VRX)_[TPO](http://www.rcsb.org/pdb/ligand/ligandsummary.do?hetId=TPO)_C, [2VRX](http://www.rcsb.org/pdb/explore.do?structureId=2VRX)_[447](http://www.rcsb.org/pdb/ligand/ligandsummary.do?hetId=447)_D, [2VRX](http://www.rcsb.org/pdb/explore.do?structureId=2VRX)_[TPO](http://www.rcsb.org/pdb/ligand/ligandsummary.do?hetId=TPO)_D | 5 | 18 |
| **Q6DE08** | 77-361 ^r^ | A/B | [2BFX](http://www.rcsb.org/pdb/explore.do?structureId=2BFX)_[TPO](http://www.rcsb.org/pdb/ligand/ligandsummary.do?hetId=TPO)_A, [2BFX](http://www.rcsb.org/pdb/explore.do?structureId=2BFX)_[TPO](http://www.rcsb.org/pdb/ligand/ligandsummary.do?hetId=TPO)_B, [2BFY](http://www.rcsb.org/pdb/explore.do?structureId=2BFY)_[H1N](http://www.rcsb.org/pdb/ligand/ligandsummary.do?hetId=H1N)_A, [2BFY](http://www.rcsb.org/pdb/explore.do?structureId=2BFY)_[TPO](http://www.rcsb.org/pdb/ligand/ligandsummary.do?hetId=TPO)_A, [2BFY](http://www.rcsb.org/pdb/explore.do?structureId=2BFY)_[H1N](http://www.rcsb.org/pdb/ligand/ligandsummary.do?hetId=H1N)_B, [2BFY](http://www.rcsb.org/pdb/explore.do?structureId=2BFY)_[TPO](http://www.rcsb.org/pdb/ligand/ligandsummary.do?hetId=TPO)_B, [2VGO](http://www.rcsb.org/pdb/explore.do?structureId=2VGO)_[AD5](http://www.rcsb.org/pdb/ligand/ligandsummary.do?hetId=AD5)_A, [2VGO](http://www.rcsb.org/pdb/explore.do?structureId=2VGO)_[TPO](http://www.rcsb.org/pdb/ligand/ligandsummary.do?hetId=TPO)_A, [2VGO](http://www.rcsb.org/pdb/explore.do?structureId=2VGO)_[AD5](http://www.rcsb.org/pdb/ligand/ligandsummary.do?hetId=AD5)_B, [2VGO](http://www.rcsb.org/pdb/explore.do?structureId=2VGO)_[TPO](http://www.rcsb.org/pdb/ligand/ligandsummary.do?hetId=TPO)_B, [2VGP](http://www.rcsb.org/pdb/explore.do?structureId=2VGP)_[AD6](http://www.rcsb.org/pdb/ligand/ligandsummary.do?hetId=AD6)_A, [2VGP](http://www.rcsb.org/pdb/explore.do?structureId=2VGP)_[TPO](http://www.rcsb.org/pdb/ligand/ligandsummary.do?hetId=TPO)_A, [2VGP](http://www.rcsb.org/pdb/explore.do?structureId=2VGP)_[AD6](http://www.rcsb.org/pdb/ligand/ligandsummary.do?hetId=AD6)_B, [2VGP](http://www.rcsb.org/pdb/explore.do?structureId=2VGP)_[TPO](http://www.rcsb.org/pdb/ligand/ligandsummary.do?hetId=TPO)_B | 4 | 14 |
|  | 78-361 ^s^ |  | [2VRX](http://www.rcsb.org/pdb/explore.do?structureId=2VRX)_[447](http://www.rcsb.org/pdb/ligand/ligandsummary.do?hetId=447)_A, [2VRX](http://www.rcsb.org/pdb/explore.do?structureId=2VRX)_[TPO](http://www.rcsb.org/pdb/ligand/ligandsummary.do?hetId=TPO)_A, [2VRX](http://www.rcsb.org/pdb/explore.do?structureId=2VRX)_[447](http://www.rcsb.org/pdb/ligand/ligandsummary.do?hetId=447)_B, [2VRX](http://www.rcsb.org/pdb/explore.do?structureId=2VRX)_[TPO](http://www.rcsb.org/pdb/ligand/ligandsummary.do?hetId=TPO)_B | 1 | 4 |
| ***Mus musculus*** | | | | | |
| **P97477** | 116-381 ^t^ | A | 3D14_AK1, 3D15_AK2, 3D2I_AK3, 3D2K_AK4, 3DAJ_FXG, 3DJ5_AK5, 3DJ6_AK6, 3DJ7_AK7 | 8 | 8 |

**Table S3.** Identification of kinases sequentially similar to AK through pairwise sequence alignment of AURKA_HUMAN against the entire kinome present in kinbase v1.1 using blast-p.

| S.  No. | Swiss-prot identifier | Gene | Uniprot identifier | Alias | Classification  (Group/Family/Sub-family) | % Identity |
| --- | --- | --- | --- | --- | --- | --- |
|  | AURKA_HUMAN | AurA | O14965 | AIK, AIRK1, ARK1, AURA, AYK1, BTAK, IAK1, STK15, STK6 | Aur | Reference  sequence |
|  | AURKB_HUMAN | AurB | Q96GD4 | AIK2, AIM1, AIRK2, ARK2, STK1, STK12, STK5 | Aur | 74 |
|  | AURKC_HUMAN | AurC | Q9UQB9 | AIE2, AIK3, AIRK3, ARK3, STK13 | Aur | 73 |
|  | KS6B2_HUMAN | p70S6Kb | Q9UBS0 | STK14B, P70-beta-1, p70S6Kb, P70-beta-2, P70-BETA, STK14B, p70(S6K)-beta, SRK, P70S6KB, RPS6KB2, KLS, S6K2, S6K-beta2 | AGC/RSK/p70 | 38 |
|  | PLK4_HUMAN | PLK4 | O00444 | SAK, STK18 | PLK/SAK | 37 |
|  | SNRK_HUMAN | SNRK | Q9NRH2 | KIAA0096, SNFRK | CAMK/CAMKL/SNRK | 36 |
|  | KS6B1_HUMAN | p70S6K | P23443 | p70(S6K)-alpha, p70s6k, S6K, STK14A, S6K1, p70S6K, RPS6KB1, PS6K, p70-alpha, p70-S6K | AGC/RSK/p70 | 36 |
|  | KS6A2_HUMAN | RSK1 | Q15349 | RPS6KA2, MAPKAPK1C, RSK3 | AGC/RSK/RSK | 35 |
|  | PDPK1_HUMAN | PDK1 | O15530 | MGC35290, PRO0461, PkB-like, PDK1, PkB-like 1, PDPK1, MGC20087 | AGC/PDK1 | 35 |
|  | MARK4_HUMAN | MARK4 | Q96L34 | KIAA1860, MARKL1 | CAMK/CAMKL/MARK | 34 |
|  | SIK3_HUMAN | QSK | Q9Y2K2 | QSK, KIAA0999, FLJ12240, L19 | CAMK/CAMKL/QIK | 34 |
|  | SIK2_HUMAN | QIK | Q9H0K1 | SNF1LK2, DKFZp434K1115, QIK, SIK2, LOH11CR1I, KIAA0781, Hs.42676 | CAMK/CAMKL/QIK | 34 |
|  | MARK1_HUMAN | MARK1 | Q9P0L2 | MGC126513, MGC126512, KIAA1477, MARK1, MARK | CAMK/CAMKL/MARK | 33 |
|  | MARK2_HUMAN | MARK2 | Q7KZI7 | EMK1, MGC99619, PAR-1, MARK2 | CAMK/CAMKL/MARK | 33 |
|  | PLK2_HUMAN | PLK2 | Q9NYY3 | PLK2, SNK | Other/PLK/PLK2 | 31 |

**Table S4.** Geometric parameters of the inter-residue metric for the identification of DFG-loop conformation in kinase based on centre of mass (COM).

| **S**  **No** | **PDB**  **id** | **Conformation** | **Distance (Å)** | | | | | |  | **Angle (°)** | | |
| --- | --- | --- | --- | --- | --- | --- | --- | --- | --- | --- | --- | --- |
|  |  | **D, C, G** | **r1**  **(GK+2···K162)** | **r2**  **(GK+2···E181)** | **r3**  **(GK+2···F275)** | **r4**  **(K162···F275)** | **r5**  **(E181···F275)** | **r6**  **(K162···E181)** |  | **∡K(GK)E** | **∡K(GK)F** | **∡E(GK)F** |
| 1 | 1OL5 | D_I_, C_I_, G_E_ | 6.656 | 7.471 | 10.021 | 9.663 | 7.787 | 3.252 |  | 25.8 | 67.4 | 50.3 |
| 2 | 2W1C | D_I_, C_I_, G_E_ | 5.789 | 7.718 | 10.494 | 10.252 | 7.516 | 5.624 |  | 46.6 | 69.6 | 45.7 |
| 3 | 1OL7 | D_I_, C_I_, G_E_ | 6.565 | 7.437 | 9.887 | 9.584 | 7.679 | 3.551 |  | 28.5 | 67.8 | 48.3 |
| 4 | 3E5A | D_I_, C_I_, G_F_ | 7.255 | 8.405 | 9.932 | 10.653 | 8.161 | 4.357 |  | 31.2 | 74.8 | 52.0 |
| 5 | 3HA6 | D_I_, C_I_, G_E_ | 6.631 | 6.943 | 9.901 | 10.671 | 7.183 | 5.292 |  | 45.8 | 79.4 | 46.5 |
| 6 | 3NRM | D_I_, C_I_, G_E_ | 6.533 | 7.228 | 9.524 | 9.079 | 8.025 | 3.836 |  | 31.9 | 65.8 | 55.2 |
| 7 | 3UO5 | D_I_, C_I_, G_E_ | 6.312 | 7.600 | 10.409 | 10.064 | 7.907 | 4.709 |  | 38.2 | 69.1 | 49.1 |
| 8 | 3UOD | D_I_, C_I_, G_E_ | 6.707 | 8.241 | 10.246 | 9.458 | 7.900 | 4.563 |  | 33.4 | 63.9 | 49.1 |
| 9 | 3UP2 | D_I_, C_I_, G_E_ | 7.543 | 8.409 | 10.433 | 10.749 | 8.039 | 4.094 |  | 29.1 | 71.4 | 49.1 |
| 10 | 3UP7 | D_I_, C_I_, G_E_ | 5.324 | 7.506 | 10.666 | 10.396 | 8.578 | 4.315 |  | 34.2 | 72.6 | 52.9 |
| 11 | 2J4Z | D_O_, C_O_, G_E_ | 6.531 | 12.301 | 7.583 | 4.675 | 4.771 | 8.040 |  | 36.5 | 37.8 | 4.2 |
| 12 | 2WTV | D_O_, C_O_, G_E_ | 7.551 | 11.837 | 6.348 | 7.549 | 5.634 | 11.108 |  | 65.5 | 65.3 | 8.6 |
| 13 | 3H10 | D_O_, C_O_, G_E_ | 7.357 | 10.032 | 6.067 | 8.208 | 4.770 | 10.724 |  | 74.0 | 72.0 | 20.2 |
| 14 | 3UNZ | D_O_, C_O_, G_E_ | 6.146 | 11.818 | 6.729 | 6.013 | 5.270 | 9.203 |  | 50.3 | 55.4 | 8.8 |
| 15 | 3UO6 | D_O_, C_O_, G_E_ | 5.989 | 13.300 | 6.802 | 5.263 | 6.412 | 11.508 |  | 59.7 | 56.6 | 5.9 |
| 16 | 3UOJ | D_O_, C_O_, G_E_ | 6.278 | 13.231 | 6.950 | 6.343 | 6.459 | 10.649 |  | 53.9 | 57.0 | 9.0 |
| 17 | 3UOK | D_O_, C_O_, G_E_ | 5.939 | 14.345 | 7.001 | 6.943 | 7.348 | 13.031 |  | 65.3 | 64.3 | 1.3 |
| 18 | 3UOL | D_O_, C_O_, G_E_ | 6.406 | 11.782 | 6.326 | 5.909 | 5.494 | 10.127 |  | 59.2 | 55.5 | 4.3 |
| 19 | 3O50 | D_O_, C_O_, G_E_ | 8.331 | 12.560 | 5.684 | 7.505 | 7.121 | 10.342 |  | 54.5 | 61.4 | 10 |
| 20 | 3FDN | D_O_, C_O_, G_E_ | 7.418 | 12.582 | 5.906 | 7.140 | 7.230 | 9.136 |  | 45.9 | 63.6 | 18.5 |
| DFG-in |  | **Min** | 5.324 | 6.943 | 9.524 | 9.079 | 7.183 | 3.252 |  | 25.8 | 63.9 | 45.7 |
|  |  | **Max** | 7.543 | 8.409 | 10.666 | 10.749 | 8.578 | 5.624 |  | 46.6 | 79.4 | 55.2 |
|  |  | **Avg** | 6.532 | 7.696 | 10.151 | 10.057 | 7.878 | 4.359 |  | 34.4 | 70.1 | 49.8 |
|  |  | **Diff** | 2.219 | 1.466 | 1.142 | 1.670 | 1.395 | 2.372 |  | 20.8 | 15.5 | 9.5 |
| DFG-out (up) | | **Min** | 5.939 | 10.032 | 5.684 | 4.675 | 4.770 | 8.040 |  | 36.5 | 37.8 | 1.3 |
|  |  | **Max** | 8.331 | 14.345 | 7.583 | 8.208 | 7.348 | 13.031 |  | 74.0 | 72.0 | 20.2 |
|  |  | **Avg** | 6.795 | 12.379 | 6.540 | 6.555 | 6.051 | 10.387 |  | 56.4 | 58.8 | 9.0 |
|  |  | **Diff** | 2.392 | 4.313 | 1.899 | 3.533 | 2.578 | 4.991 |  | 37.5 | 34.2 | 18.9 |
|  |  |  |  | ★ | ★★ | ★★ |  | ★★ |  |  |  | ★★ |

**Table S5.** Performance of the inter-residue metric based on centre of mass (COM) in identifying the DFG-loop conformation of AK.

| **S No** | **PDB id** | **Distance (Å)** | | | | | |  | **Angle (°)** | | |  | **Conformation** | |
| --- | --- | --- | --- | --- | --- | --- | --- | --- | --- | --- | --- | --- | --- | --- |
|  |  | **r1**  **(GK …K162)** | **r2**  **(GK …E181)** | **r3**  **(GK …F275)** | **r4**  **(K162 …F275)** | **r5**  **(E181 …F275)** | **r6**  **(K162 …E181)** |  | **∡K(GK)E** | **∡K(GK)F** | **∡E(GK)F** |  | **Observed** | **Predicted** |
| 1 | 1OL6 | 7.055 | 8.739 | 10.351 | 10.760 | 8.820 | 4.573 |  | 31.4 | 73.6 | 54.2 |  | DFG-in | DFG-in |
| 2 | 1MQ4 | 6.763 | 7.217 | 10.293 | 9.483 | 7.868 | 3.252 |  | 26.7 | 63.7 | 49.7 |  | DFG-in | DFG-in |
| 3 | 2DWB | 6.808 | 7.439 | 10.215 | 9.462 | 8.038 | 3.608 |  | 28.9 | 63.9 | 51.3 |  | DFG-in | DFG-in |
| 4 | 2W1D | 5.676 | 7.203 | 10.129 | 9.975 | 7.394 | 5.383 |  | 48 | 72.1 | 46.9 |  | DFG-in | DFG-in |
| 5 | 2W1E | 6.025 | 7.588 | 10.354 | 9.702 | 6.767 | 5.431 |  | 45.2 | 66.7 | 40.8 |  | DFG-in | DFG-in |
| 6 | 2W1F | 6.448 | 7.068 | 10.416 | 10.593 | 7.317 | 5.532 |  | 47.8 | 72.3 | 44.6 |  | DFG-in | DFG-in |
| 7 | 2W1G | 5.713 | 7.361 | 10.530 | 10.503 | 7.982 | 5.372 |  | 46.4 | 72.2 | 49.2 |  | DFG-in | DFG-in |
| 8 | 2WTW | 6.633 | 9.281 | 10.207 | 10.790 | 7.094 | 6.625 |  | 42.4 | 76.4 | 40.9 |  | DFG-in | DFG-in |
| 9 | 2X6D | 5.390 | 7.257 | 9.805 | 9.728 | 7.338 | 3.962 |  | 32.4 | 73.2 | 49 |  | DFG-in | DFG-in |
| 10 | 2XNE | 6.742 | 7.852 | 10.077 | 9.717 | 7.754 | 5.122 |  | 40.2 | 68 | 49.2 |  | DFG-in | DFG-in |
| 11 | 2XNG | 6.463 | 7.461 | 10.229 | 9.832 | 7.710 | 4.683 |  | 38.5 | 68 | 48.6 |  | DFG-in | DFG-in |
| 12 | 2XRU | 5.459 | 6.816 | 10.383 | 10.412 | 7.467 | 4.787 |  | 44.2 | 75.1 | 45.9 |  | DFG-in | DFG-in |
| 13 | 3MYG | 6.249 | 6.156 | 10.287 | 9.688 | 8.118 | 3.113 |  | 29.1 | 66.6 | 52.1 |  | DFG-in | DFG-in |
| 14 | 1MUO | 5.548 | 11.282 | 5.309 | 3.011 | 6.419 | 8.454 |  | 46.6 | 30.6 | 17.5 |  | DFG-out (up) | DFG-out (up) |
| 15 | 2BMC | 6.723 | 14.000 | 5.820 | 6.262 | 7.216 | 10.245 |  | 52.6 | 59.4 | 11.6 |  | DFG-out (up) | DFG-out (up) |
| 16 | 2C6D | 5.666 | 14.995 | 12.361 | 10.272 | 7.279 | 13.274 |  | 61.7 | 55.6 | 28.90 |  | DFG-out (up) | DFG-out (up) |
| 17 | 2J50 | 6.992 | 12.542 | 5.883 | 4.080 | 7.167 | 9.779 |  | 50.9 | 36 | 17.8 |  | DFG-out (up) | DFG-out (up) |
| 18 | 3H0Y | 7.594 | 12.371 | 6.376 | 7.017 | 6.204 | 9.202 |  | 48.6 | 60.5 | 13.1 |  | DFG-out (up) | DFG-out (up) |
| 19 | 3K5U | 6.960 | 12.456 | 6.027 | 7.308 | 7.010 | 9.664 |  | 50.5 | 68 | 18.6 |  | DFG-out (up) | DFG-out (up) |
| 20 | 2NP8 | 6.941 | 7.081 | 11.130 | 10.834 | 6.358 | 6.193 |  | 51.7 | 69.3 | 31.8 |  | DFG-in | DFG-out |
| 21 | 2C6E | 7.356 | 12.506 | 9.060 | 5.599 | 11.958 | 11.279 |  | 63.0 | 38.0 | 65.00 |  | DFG-out | DFG-out |
| 22 | 3H0Z | 6.880 | 8.328 | 11.409 | 10.542 | 4.899 | 7.271 |  | 56.2 | 22.5 | 22.5 |  | DFG-out | DFG-out |

* Black: DFG-in measure; Red: DFG-out (up); Grey: Overlapping, ambiguous

**Table S6.** Prioritizing the parameters of the inter-motif metric based on their performance in distinguishing the DFG-conformation of AK.

| **Inter-motif** | | **TRUE** | **T (%)** |  | **ERROR** | **E (%)** | **Weights** |  | **Weighted Parameters** | | |
| --- | --- | --- | --- | --- | --- | --- | --- | --- | --- | --- | --- |
| **1Parameter Equation** | |  |  |  |  |  |  |  | **5** |  |  |
| P1 | r1(GK …K162) | 60 | 1.5 |  | 3940 | 98.5 |  |  | P9 ★★ | ∡3 (E...GK...F) | |
| P2 | r2(GK …E181) | 3816 | 95.4 |  | 276 | 6.9 | ★ |  | P3★★ | r3(GK …F275) | |
| P3 | r3(GK …F275) | 3988 | 99.7 |  | 12 | 0.3 | ★★ |  | P6★ | r6(K162 …E181) | |
| P4 | r4(K162 …F275) | 3724 | 93.1 |  | 184 | 4.6 | ★ |  | P2★ | r2(GK …E181) | |
| P5 | r5(E181 …F275) | 2192 | 54.8 |  | 1808 | 45.2 |  |  | P4★ | r4(K162 …F275) | |
| P6 | r6(K162 …E181) | 3848 | 96.2 |  | 152 | 3.8 | ★ |  |  |  |  |
| P7 | ∡1 (K...GK...E) | 1816 | 45.4 |  | 1048 | 26.2 |  |  | Average Prediction Accuracy of ALL parameters | 67.73% | 32.27 |
| P8 | ∡2 (K...GK...F) | 944 | 23.6 |  | 460 | 11.5 |  |  | Average Prediction Accuracy of weighted parameters | **96.86%** | **3.14%** |
| P9 | ∡3 ([E...GK...F) | 3996 | 99.9 |  | 4 | 0.1 | ★★ |  |  |  |  |
| **2Parameter Equation** | |  |  |  |  |  |  |  | **4** |  |  |
| P1′ | r2+r3 | 7804 | 97.55 |  | 196 | 2.45 |  |  | P9 ★★ | ∡3 (E...GK...F) | |
| P2′ | r2+r4 | 7540 | 94.25 |  | 460 | 5.75 |  |  | P3★★ | r3(GK …F275) | |
| P3′ | r2+r6 | 7664 | 95.8 |  | 336 | 4.2 |  |  | P6★ | r6(K162 …E181) | |
| P4′ | r2+∡3 | 7812 | 97.65 |  | 188 | 2.35 | ★ |  | P2★ | r2(GK …E181) | |
| P5′ | r3+r4 | 7712 | 96.4 |  | 288 | 3.6 |  |  |  |  |  |
| P6′ | r3+r6 | 7836 | 97.95 |  | 164 | 2.05 | ★ |  | Average Prediction Accuracy of ALL parameters | 96.86% | 3.14% |
| P7′ | r3+∡3 | 7984 | 99.8 |  | 16 | 0.2 | ★★ |  | Average Prediction Accuracy of weighted parameters | **98.36%** | **1.64%** |
| P8′ | r4+r6 | 7572 | 94.65 |  | 428 | 5.35 |  |  |  |  |  |
| P9′ | r4+∡3 | 7720 | 96.5 |  | 280 | 3.5 |  |  |  |  |  |
| **P10′** | r6+∡3 | 7844 | 98.05 |  | 156 | 1.95 | ★★ |  |  |  |  |
| **3Parameter Equation** | |  |  |  |  |  |  |  | **4** |  |  |
| P1′′ | r3+r2+r6 | 11652 | 97.10 |  | 348 | 2.90 |  |  | P9 ★★ | ∡3 (E...GK...F) | |
| P2′′ | r3+r2+∡3 | 11800 | 98.33 |  | 200 | 1.67 |  |  | P3★★ | r3(GK …F275) | |
| P3′′ | r3+r6+∡3 | 11832 | 98.60 |  | 168 | 1.40 | ★ |  | P6★ | r6(K162 …E181) | |
| P4′′ | r4+r6+∡3 | 11568 | 96.40 |  | 432 | 3.60 | ★★ |  | P2★ | r2(GK …E181) | |
|  |  |  |  |  |  |  |  |  |  |  |  |
|  |  |  |  |  |  |  |  |  | Average Prediction Accuracy of ALL parameters | 97.80% | 2.20% |
|  |  |  |  |  |  |  |  |  | Average Prediction Accuracy of weighted parameters | **98.46%** | **1.54%** |

**Table S7.** Geometric parameters of the intra-motif DGF- and A-loop metric for the identification of DFG-loop conformation in kinase based on centre of mass (COM).

| **S**  **No** | **PDB**  **id** | **Conformation**  **D, C, G** | **Distance (Å)** | | | | | |  | **Angle (°)** | | |
| --- | --- | --- | --- | --- | --- | --- | --- | --- | --- | --- | --- | --- |
|  |  |  | **r1**  **(A273...D274)** | **r2**  **(D274...F275)** | **r3**  **(F275-G276)** | **r4**  **(D274...G276)** | **r5**  **(F275-W277)** | **r6**  **(F275...T288)** |  | **∡DFG** | **∡FWH** | **∡FWT** |
| 1 | 1OL5 | D_I_, C_I_, G_E_ | 5.890 | 8.197 | 7.585 | 5.357 | 11.770 | 16.323 |  | 39.4 | 43.8 | 102.0 |
| 2 | 2W1C | D_I_, C_I_, G_E_ | 5.356 | 7.717 | 7.416 | 5.659 | 12.478 | 19.981 |  | 43.9 | 42.5 | 168.8 |
| 3 | 1OL7 | D_I_, C_I_, G_E_ | 5.887 | 8.168 | 7.636 | 5.474 | 12.275 | 19.208 |  | 41.2 | 42.9 | 168.5 |
| 4 | 3E5A | D_I_, C_I_, G_F_ | 5.781 | 7.888 | 7.765 | 5.266 | 12.250 | 15.699 |  | 39.3 | 47.4 | 109.2 |
| 5 | 3HA6 | D_I_, C_I_, G_E_ | 4.489 | 8.114 | 7.673 | 8.174 | 12.087 | 16.160 |  | 40.7 | 47.1 | 108.8 |
| 6 | 3NRM | D_I_, C_I_, G_E_ | 4.385 | 8.668 | 7.727 | 8.179 | 11.736 | 14.765 |  | 39.5 | 38.9 | 124.6 |
| 7 | 3UO4 | D_I_, C_I_, G_E_ | 5.658 | 8.106 | 7.470 | 5.266 | 12.029 | 19.945 |  | 41.4 | 46.5 | 159.6 |
| 8 | 3UOD | D_I_, C_I_, G_E_ | 5.566 | 8.060 | 7.394 | 5.488 | 11.821 | 18.535 |  | 42.1 | 42.6 | 153.3 |
| 9 | 3UP2 | D_I_, C_I_, G_E_ | 5.570 | 7.683 | 7.645 | 5.189 | 12.066 | 19.920 |  | 37.4 | 42.3 | 149.7 |
| 10 | 3UP7 | D_I_, C_I_, G_E_ | 4.242 | 8.546 | 7.485 | 7.944 | 12.233 | 19.481 |  | 49.0 | 56.0 | 161.0 |
| 11 | 2J4Z | D_O_, C_O_, G_E_ | 5.510 | 8.637 | 5.068 | 7.439 | 5.525 | 21.306 |  | 59.1 | 67.7 | 109.7 |
| 12 | 2WTV | D_O_, C_O_, G_E_ | 4.625 | 5.385 | 7.768 | 8.820 | 6.507 | 20.496 |  | 86.4 | 124.7 | 121.6 |
| 13 | 3H10 | D_O_, C_O_, G_E_ | 4.638 | 5.375 | 7.720 | 9.423 | 6.269 | 20.406 |  | 90.2 | 112.9 | 136.0 |
| 14 | 3UNZ | D_O_, C_O_, G_E_ | 5.199 | 8.070 | 5.768 | 7.414 | 7.812 | 21.322 |  | 62.2 | 100.1 | 114.8 |
| 15 | 3UO6 | D_O_, C_O_, G_E_ | 5.233 | 8.380 | 5.483 | 7.067 | 7.612 | 22.049 |  | 58.7 | 106.0 | 117.3 |
| 16 | 3UOJ | D_O_, C_O_, G_E_ | 5.408 | 7.942 | 4.983 | 6.924 | 7.533 | 21.970 |  | 59.7 | 109.1 | 117.0 |
| 17 | 3UOK | D_O_, C_O_, G_E_ | 5.281 | 7.663 | 5.203 | 7.002 | 7.715 | 21.377 |  | 62.5 | 102.5 | 120.7 |
| 18 | 3UOL | D_O_, C_O_, G_E_ | 5.936 | 7.841 | 5.029 | 7.591 | 7.889 | 21.980 |  | 68.9 | 97.7 | 112.1 |
| 19 | 3UO5 | D_O_, C_O_, G_E_ | 6.153 | 8.281 | 7.777 | 5.286 | 9.736 | 21.488 |  | 62.3 | 98.4 | 115.6 |
| 20 | 3FDN | D_O_, C_O_, G_E_ | 5.546 | 7.746 | 7.879 | 3.999 | 7.803 | 21.930 |  | 69.6 | 127.2 | 112.2 |
|  | **DFG-in** | **Min** | 4.242 | 7.683 | 7.394 | 5.189 | 11.736 | 14.765 |  | 37.4 | 38.9 | 102.0 |
|  |  | **Max** | 5.890 | 8.668 | 7.765 | 8.179 | 12.478 | 19.981 |  | 49.0 | 56.0 | 168.8 |
|  |  | **Avg** | 5.282 | 8.115 | 7.580 | 6.200 | 12.075 | 18.002 |  | 41.4 | 45.0 | 140.6 |
|  |  | **Diff** | 1.648 | 0.985 | 0.371 | 2.990 | 0.742 | 5.216 |  | 11.6 | 17.1 | 66.8 |
|  | **DFG-out (up)** | **Min** | 4.625 | 5.375 | 4.983 | 3.999 | 5.525 | 20.406 |  | 58.7 | 67.7 | 109.7 |
|  |  | **Max** | 6.153 | 8.637 | 7.879 | 9.423 | 9.736 | 22.049 |  | 90.2 | 127.2 | 136.0 |
|  |  | **Avg** | 5.353 | 7.532 | 6.268 | 7.097 | 7.440 | 21.432 |  | 68.0 | 104.6 | 117.7 |
|  |  | **Diff** | 1.528 | 3.262 | 2.896 | 5.424 | 4.211 | 1.643 |  | 31.5 | 59.5 | 26.3 |
|  |  |  |  |  |  |  | ★ | ★ |  | ★★ | ★★ |  |

**Table S8.** Performance of the intra-residue DFG- and A-loop metric based on centre of mass (COM) in identifying the DFG-loop conformation of AK.

| **S**  **No** | **PDB**  **id** | **Distance (Å)** | | | | | |  | **Angle (°)** | | |  | **Conformation** | |
| --- | --- | --- | --- | --- | --- | --- | --- | --- | --- | --- | --- | --- | --- | --- |
|  |  | **r1**  **(A273...D274)** | **r2**  **(D274...F275)** | **r3**  **(F275-G276)** | **r4**  **(D274...G276)** | **r5**  **(F275-W277)** | **r6**  **(F275...T288)** |  | **∡DFG** | **∡FWH** | **∡FWT** |  | **Observed** | **Predicted** |
| 1 | 1OL6 | 4.231 | 9.303 | 7.712 | 7.904 | 12.101 | 14.391 |  | 49.5 | 44.5 | 128.6 |  | DFG-in | DFG-in |
| 2 | 1MQ4 | 5.836 | 8.198 | 7.621 | 5.849 | 12.078 | 16.852 |  | 42.1 | 43.8 | 138.9 |  | DFG-in | DFG-in |
| 3 | 2DWB | 5.786 | 4.875 | 7.731 | 5.185 | 12.117 | 17.150 |  | 39.4 | 45.0 | 142.5 |  | DFG-in | DFG-in |
| 4 | 2W1D | 5.078 | 7.579 | 7.381 | 6.341 | 12.333 | 15.821 |  | 49.1 | 46.3 | 136.9 |  | DFG-in | DFG-in |
| 5 | 2W1E | 5.222 | 7.630 | 7.587 | 5.806 | 12.261 | 16.453 |  | 44.9 | 54.7 | 126.5 |  | DFG-in | DFG-in |
| 6 | 2W1F | 5.722 | 7.704 | 7.535 | 5.185 | 12.580 | 17.821 |  | 39.8 | 39.6 | 143.2 |  | DFG-in | DFG-in |
| 7 | 2W1G | 5.940 | 8.024 | 7.479 | 4.999 | 12.474 | 15.769 |  | 37.4 | 39.9 | 128.4 |  | DFG-in | DFG-in |
| 8 | 2WTW | 4.062 | 8.025 | 7.653 | 4.683 | 11.896 | 18.685 |  | 37.1 | 46.3 | 116.2 |  | DFG-in | DFG-in |
| 9 | 2X6D | 5.489 | 7.871 | 7.618 | 5.530 | 12.354 | 16.980 |  | 41.8 | 61.7 | 166.3 |  | DFG-in | DFG-in |
| 10 | 2XNE | 4.325 | 7.817 | 7.718 | 7.834 | 12.567 | 15.235 |  | 38.1 | 40.7 | 119.7 |  | DFG-in | DFG-in |
| 11 | 2XNG | 5.648 | 8.371 | 7.534 | 5.346 | 10.050 | 17.983 |  | 38.8 | 50.1 | 123.9 |  | DFG-in | DFG-in |
| 12 | 2XRU | 4.804 | 7.979 | 7.331 | 6.700 | 11.413 | 18.792 |  | 41.6 | 54.5 | 139.1 |  | DFG-in | DFG-in |
| 13 | 3MYG | 4.456 | 8.623 | 7.672 | 7.994 | 11.607 | 16.908 |  | 48.4 | 45.0 | 145.2 |  | DFG-in | DFG-in |
| 14 | 1MUO | 4.727 | 7.452 | 7.408 | 9.660 | 6.547 | 21.563 |  | 81.1 | 96.5 | 122.5 |  | DFG-out (up) | DFG-out (up) |
| 15 | 2BMC | 5.078 | 7.548 | 7.697 | 4.790 | 7.503 | 22.896 |  | 66.6 | 108.9 | 133.4 |  | DFG-out (up) | DFG-out (up) |
| 16 | 2C6D | 4.723 | 8.170 | 6.513 | 5.658 | 7.492 | 21.356 |  | 79.9 | 111.8 | 123.0 |  | DFG-out (up) | DFG-out (up) |
| 17 | 2J50 | 4.951 | 7.070 | 7.849 | 4.623 | 8.941 | 20.675 |  | 64.2 | 112.6 | 133.7 |  | DFG-out (up) | DFG-out (up) |
| 18 | 3H0Y | 4.930 | 7.956 | 6.818 | 7.461 | 11.820 | 22.612 |  | 58.4 | 103.4 | 129.4 |  | DFG-out (up) | DFG-out (up) |
| 19 | 3K5U | 4.893 | 7.757 | 7.827 | 4.915 | 7.454 | 21.786 |  | 56.8 | 127.1 | 124.2 |  | DFG-out (up) | DFG-out (up) |
| 20 | 2NP8 | 4.52 | 7.554 | 7.329 | 4.758 | 11.654 | 20.100 |  | 33.5 | 29.2 | 118.9 |  | DFG-in | DFG-out |
| 21 | 2C6E | 4.277 | 7.847 | 7.344 | 5.899 | 10.499 | 21.488 |  | 62.3 | 98.4 | 115.6 |  | DFG-out | DFG-out |
| 22 | 3H0Z | 4.009 | 8.242 | 7.239 | 7.514 | 9.107 | 20.985 |  | 58.3 | 55.9 | 145.5 |  | DFG-out | DFG-out |

* Black: DFG-in measure; Orange: DFG-out (up); Grey: Overlapping, ambiguous

**Table S9.** Prioritizing the parameters of the intra DFG- and A-loop motif metric based on their performance in distinguishing the DFG conformation of AK.

| **Intra-DFG-Aloop-motif** | | **TRUE** | **T(%)** |  | **ERROR** | **E(%)** | **Weights** |  | **Weighted Parameters** | | |
| --- | --- | --- | --- | --- | --- | --- | --- | --- | --- | --- | --- |
| 1Parameter Equation | |  |  |  |  |  |  |  | **4** |  |  |
| P1 | r1 (A273...D274) | 44 | 1.1 |  | 3956 | 98.9 |  |  | P8★ | ∡2 (FWH) | |
| P2 | r2 (D274...F275) | 36 | 0.9 |  | 3964 | 99.1 |  |  | P7★ | ∡1 (DFG) | |
| P3 | r3 (F275-G276) | 72 | 1.8 |  | 3928 | 98.2 |  |  | P5★ | r5 (F275-W277) | |
| P4 | r4 (D274...G276) | 52 | 1.3 |  | 3948 | 98.7 |  |  | P6★ | r6 (F275...T288) | |
| P5 | r5 (F275-W277) | 3812 | 95.3 |  | 188 | 4.7 | ★ |  |  |  |  |
| P6 | r6 (F275...T288) | 3684 | 92.1 |  | 316 | 7.9 | ★ |  | Avg Prediction Accuracy of ALL parameters | 43.6% | 56.4% |
| P7 | ∡1 (DFG) | 3912 | 97.8 |  | 88 | 2.2 | ★★ |  | Avg Prediction Accuracy of weighted parameters | **96.2%** | **3.8%** |
| P8 | ∡2 (FWH) | 3984 | 99.6 |  | 16 | 0.4 | ★★ |  |  |  |  |
| P9 | ∡3 (FWT) | 88 | 2.2 |  | 3912 | 97.8 |  |  |  |  |  |
| 2Parameter Equation | |  |  |  |  |  |  |  | **3** |  |  |
| P1′ | ∡2 +∡1 | 7896 | 98.7 |  | 104 | 1.3 | ★★ |  | P8★ | ∡2 (FWH) | |
| P2′ | ∡2 + r5 | 7796 | 97.45 |  | 204 | 2.55 | ★ |  | P7★ | ∡1 (DFG) | |
| P3′ | ∡2 + r6 | 7668 | 95.85 |  | 332 | 4.15 |  |  | P5★ | r5 (F275-W277) | |
| P4′ | ∡1 + r5 | 7724 | 96.55 |  | 276 | 3.45 |  |  |  |  |  |
| P5′ | ∡1 + r6 | 7596 | 94.95 |  | 404 | 5.05 |  |  | Avg Prediction Accuracy of ALL parameters | 96.2% | 3.8% |
| P6′ | r5+r6 | 7496 | 93.7 |  | 504 | 6.3 |  |  | Avg Prediction Accuracy of weighted parameters | **98.1%** | **1.92%** |
| 3Parameter Equation | |  |  |  |  |  |  |  | **2** |  |  |
| P1′′ | ∡2 +∡1+r5 | 11708 | 97.6 |  | 292 | 2.4 | ★★ |  | P8★ | ∡2 (FWH) | |
|  |  |  |  |  |  |  |  |  | P7★ | ∡1 (DFG) | |
|  |  |  |  |  |  |  |  |  |  |  | |
|  |  |  |  |  |  |  |  |  |  |  |  |
|  |  |  |  |  |  |  |  |  | Avg Prediction Accuracy of ALL parameters | 97.6% | 2.4% |
|  |  |  |  |  |  |  |  |  | Avg Prediction Accuracy of weighted parameters | **97.6%** | **2.4%** |

**Table S11.** Interacting chemotypes of AK co-crystals present in Protein Data Bank (PDB).

* Blue colour represents sub-structures which preferentially bind to the DFG-in while the peach coloured ones prefer the non DFG-in conformations.

| **S.**  **No.** | **PDB**  **ID** | **Ligand ID** | **Protein-Ligand Interactions** | |
| --- | --- | --- | --- | --- |
| **1.** | [**1MQ4**](http://www.rcsb.org/pdb/explore.do?structureId=1MQ4) | [**ADP**](http://www.rcsb.org/pdb/ligand/ligandsummary.do?hetId=ADP) | O2B(ADP) : N(K163) 2.63  O1B(ADP) : NZ(K162) 2.89  O1A(ADP) : NZ(K162) 2.64  O3B(ADP) : OD2(D274) 4.37  O2A(ADP) : OD2(D274) 4.26  O2A(ADP) : OD1(N261) 2.96  O3(ADP) : O(E260) 2.60  N1(ADP) : N(A213) 3.17  N6(ADP) : O(E211) 2.70 |  |
|  |  | [**MG**](http://www.rcsb.org/pdb/ligand/ligandsummary.do?hetId=MG) |  |  |
|  |  | [**PO4**](http://www.rcsb.org/pdb/ligand/ligandsummary.do?hetId=PO4) |  |  |
| **2.** | [**1MUO**](http://www.rcsb.org/pdb/explore.do?structureId=1MUO) | [**ADN**](http://www.rcsb.org/pdb/ligand/ligandsummary.do?hetId=ADN) | O5(ADN) : NE1(W277) 2.95  N(ADN) : O(E211) 2.63  N1(ADN) : N(A213) 2.62 |  |
| **3.** | [**1OL5**](http://www.rcsb.org/pdb/explore.do?structureId=1OL5) | [**ADP**](http://www.rcsb.org/pdb/ligand/ligandsummary.do?hetId=ADP) | O1A(ADP) : NZ(K162) 2.87  O3(ADP) : O(E260) 2.68  N6(ADP) : O(E211) 3.03 |  |
|  |  | [**MG**](http://www.rcsb.org/pdb/ligand/ligandsummary.do?hetId=MG) |  |  |
|  |  | [**SO4**](http://www.rcsb.org/pdb/ligand/ligandsummary.do?hetId=SO4) |  |  |
|  |  | [**TPO**](http://www.rcsb.org/pdb/ligand/ligandsummary.do?hetId=TPO) |  |  |
| **4.** | [**1OL6**](http://www.rcsb.org/pdb/explore.do?structureId=1OL6) | [**ATP**](http://www.rcsb.org/pdb/ligand/ligandsummary.do?hetId=ATP) | O1B(ATP) : N(K143) 2.46  O3G (ATP) : NZ(K162) 2.71  O1A(ATP) : NZ(K162) 2.95  N1(ATP) : N(A213) 3.31  N6(ATP) : O(E211) 2.86 |  |
| **5.** | [**1OL7**](http://www.rcsb.org/pdb/explore.do?structureId=1OL7) | [**ADP**](http://www.rcsb.org/pdb/ligand/ligandsummary.do?hetId=ADP) | O2B(ADP) : OD1(D274) 2.84  O1A(ADP) : NZ(K162) 2.67  O3(ADP) : O(E260) 2.82  O2A(ADP) : OD1(N261) 3.09 |  |
|  |  | [**MG**](http://www.rcsb.org/pdb/ligand/ligandsummary.do?hetId=MG) |  |  |
|  |  | [**TPO**](http://www.rcsb.org/pdb/ligand/ligandsummary.do?hetId=TPO) |  |  |
| **6.** | [**2BMC**](http://www.rcsb.org/pdb/explore.do?structureId=2BMC) | [**MPY**](http://www.rcsb.org/pdb/ligand/ligandsummary.do?hetId=MPY) | O26(MPY) : NZ(K162) 3.21  N4(MPY) : N(A213) 2.78 |  |
| **7.** | [**2C6D**](http://www.rcsb.org/pdb/explore.do?structureId=2C6D) | [**ANP**](http://www.rcsb.org/pdb/ligand/ligandsummary.do?hetId=ANP) | O2B(ANP) : OG(S277) 3.20  O3A(ANP) : OG(S277) 3.30  O1A(ANP) : N(V278) 2.77  O4(ANP) : NZ(K161) 3.66  N6(ANP) : O(E210) 2.91  N1(ANP) : N(A212) 3.19 |  |
|  |  | [**GOL**](http://www.rcsb.org/pdb/ligand/ligandsummary.do?hetId=GOL) |  |  |
|  |  | [**PO4**](http://www.rcsb.org/pdb/ligand/ligandsummary.do?hetId=PO4) |  |  |
| **8.** | [**2C6E**](http://www.rcsb.org/pdb/explore.do?structureId=2C6E) | [**HPM**](http://www.rcsb.org/pdb/ligand/ligandsummary.do?hetId=HPM) | N17(HPM) : N(A212) 3.12  N24(HPM) : NZ(K161) 3.10  O36(HPM) : NZ(K161)2.76 |  |
| **9.** | [**2DWB**](http://www.rcsb.org/pdb/explore.do?structureId=2DWB) | [**ANP**](http://www.rcsb.org/pdb/ligand/ligandsummary.do?hetId=ANP) | O1B(ANP) : N(K143) 2.89  O2B(ANP) : NZ(K162) 2.83  O1A(ANP) : NZ(K162) 2.81  N6(ANP) : O(E211) 2.78  N1(ANP) : N(A213) 3.15 |  |
|  |  | [**MSE**](http://www.rcsb.org/pdb/ligand/ligandsummary.do?hetId=MSE) |  |  |
|  |  | [**SO4**](http://www.rcsb.org/pdb/ligand/ligandsummary.do?hetId=SO4) |  |  |
| **10.** | [**2J4Z**](http://www.rcsb.org/pdb/explore.do?structureId=2J4Z) | [**626**](http://www.rcsb.org/pdb/ligand/ligandsummary.do?hetId=626) | NH(626) : O(E211) 2.74  N6(626) : N(A213) 2.85  N7(626) : O(A213) 3.17 |  |
|  |  | [**ARS**](http://www.rcsb.org/pdb/ligand/ligandsummary.do?hetId=ARS) |  |  |
| **11.** | [**2J50**](http://www.rcsb.org/pdb/explore.do?structureId=2J50) | [**627**](http://www.rcsb.org/pdb/ligand/ligandsummary.do?hetId=627) | O34(627) : NZ(K162) 2.55  N4(627) : N(A213) 2.76 |  |
|  |  | [**SO4**](http://www.rcsb.org/pdb/ligand/ligandsummary.do?hetId=SO4) |  |  |
| **12.** | [**2NP8**](http://www.rcsb.org/pdb/explore.do?structureId=2NP8) | [**CC3**](http://www.rcsb.org/pdb/ligand/ligandsummary.do?hetId=CC3) | O27(CC3) : NH2(R137) 3.23  N25(CC3) : O(P214) 3.28  N18(CC3) : O(A213) 2.68  N15(CC3) : N(A213) 2.83 | c |
|  |  | [**SO4**](http://www.rcsb.org/pdb/ligand/ligandsummary.do?hetId=SO4) |  |  |
| **13.** | [**2W1C**](http://www.rcsb.org/pdb/explore.do?structureId=2W1C) | [**L0C**](http://www.rcsb.org/pdb/ligand/ligandsummary.do?hetId=L0C) | N13(L0C) : O(E211) 3.01  N15(L0C) : N(A213) 3.07  N24(L0C) : O(P214) 3.21 |  |
|  |  | [**TPO**](http://www.rcsb.org/pdb/ligand/ligandsummary.do?hetId=TPO) |  |  |
| **14.** | [**2W1D**](http://www.rcsb.org/pdb/explore.do?structureId=2W1D) | [**L0D**](http://www.rcsb.org/pdb/ligand/ligandsummary.do?hetId=L0D) | N5(L0D) : O(E211) 3.00  N4(L0D) : N(A213) 3.10  N15(L0D) : O(A213) 3.02 |  |
| **15.** | [**2W1E**](http://www.rcsb.org/pdb/explore.do?structureId=2W1E) | [**L0E**](http://www.rcsb.org/pdb/ligand/ligandsummary.do?hetId=L0E) | N6(L0E) : O(E211) 2.87  N8(L0E) : N(A213) 3.07  N26(L0E) : O(A213) 2.87 |  |
| **16.** | [**2W1F**](http://www.rcsb.org/pdb/explore.do?structureId=2W1F) | [**L0F**](http://www.rcsb.org/pdb/ligand/ligandsummary.do?hetId=L0F) | N6(L0F) : O(E211) 3.03  N8(L0F) : N(E211) 3.13  N18(L0F) : O(A213) 2.95 |  |
| **17.** | [**2W1G**](http://www.rcsb.org/pdb/explore.do?structureId=2W1G) | [**L0G**](http://www.rcsb.org/pdb/ligand/ligandsummary.do?hetId=L0G) | N10(L0G) : O(E211) 3.08  N12(L0G) : N(A213) 3.19  N15(L0G) : O(A213) 2.81 |  |
|  |  | [**TPO**](http://www.rcsb.org/pdb/ligand/ligandsummary.do?hetId=TPO) |  |  |
| **20.** | [**2WQE**](http://www.rcsb.org/pdb/explore.do?structureId=2WQE) | [**ADP**](http://www.rcsb.org/pdb/ligand/ligandsummary.do?hetId=ADP) | O3B(ADP) : N(K143) 3.27  O2A(ADP) : NZ(K162) 3.10  N1(ADP) : N(A213) 2.94  N6(ADP) : O(E211) 2.59 |  |
| **21.** | [**2WTV**](http://www.rcsb.org/pdb/explore.do?structureId=2WTV) | [**ACT**](http://www.rcsb.org/pdb/ligand/ligandsummary.do?hetId=ACT) |  |  |
|  |  | [**CME**](http://www.rcsb.org/pdb/ligand/ligandsummary.do?hetId=CME) |  |  |
|  |  | [**DMS**](http://www.rcsb.org/pdb/ligand/ligandsummary.do?hetId=DMS) |  |  |
|  |  | [**EDO**](http://www.rcsb.org/pdb/ligand/ligandsummary.do?hetId=EDO) |  |  |
|  |  | [**TPO**](http://www.rcsb.org/pdb/ligand/ligandsummary.do?hetId=TPO) |  |  |
|  |  | [**ZZL**](http://www.rcsb.org/pdb/ligand/ligandsummary.do?hetId=ZZL) | N10(ZZL) : N(A213) 2.90  N8(ZZL) : O(A213) 2.82 |  |
| **22.** | [**2WTW**](http://www.rcsb.org/pdb/explore.do?structureId=2WTW) | [**ZZL**](http://www.rcsb.org/pdb/ligand/ligandsummary.do?hetId=ZZL) | N8(ZZL) : O(A213) 2.88  N10(ZZL) : N(A213) 2.70 |  |
| **23.** | [**2X6D**](http://www.rcsb.org/pdb/explore.do?structureId=2X6D) | [**SO4**](http://www.rcsb.org/pdb/ligand/ligandsummary.do?hetId=SO4) |  |  |
| **24.** | [**2X6D**](http://www.rcsb.org/pdb/explore.do?structureId=2X6D) | [**X6D**](http://www.rcsb.org/pdb/ligand/ligandsummary.do?hetId=X6D) | NAX(X6D) : O(A213) 2.88  NAV(X6D) : N(A213) 2.89 |  |
| **25.** | [**2X6E**](http://www.rcsb.org/pdb/explore.do?structureId=2X6E) | [**YM4**](http://www.rcsb.org/pdb/ligand/ligandsummary.do?hetId=YM4) | N14(YM4) : N(A213) 2.74 |  |
| **26.** | [**2X81**](http://www.rcsb.org/pdb/explore.do?structureId=2X81) | [**ZZL**](http://www.rcsb.org/pdb/ligand/ligandsummary.do?hetId=ZZL) | N10(ZZL) : N(A213) 3.06  N8(ZZL) : O(A213) 2.80  O1(ZZL) : NH1(R137) 3.14  NH2(ZZL) : NH2(R137) 2.62 |  |
| **27.** | [**2XNE**](http://www.rcsb.org/pdb/explore.do?structureId=2XNE) | [**ASH**](http://www.rcsb.org/pdb/ligand/ligandsummary.do?hetId=ASH) | N4(ASH) : N(A213) 2.96  N17(ASH) : O(A213) 2.83 |  |
| **28.** | [**2XNG**](http://www.rcsb.org/pdb/explore.do?structureId=2XNG) | [**A0H**](http://www.rcsb.org/pdb/ligand/ligandsummary.do?hetId=A0H) | N4(AOH) : N(A213) 2.93  N23(AOH) : O(A213) 3.25  O15(AOH) : OG31(T217) 4.07 |  |
| **29.** | [**2XRU**](http://www.rcsb.org/pdb/explore.do?structureId=2XRU) | [**400**](http://www.rcsb.org/pdb/ligand/ligandsummary.do?hetId=400) | O26(400) : NZ(K162) 3.20  N3(400) : O(E211) 2.70  O15(400) : N(A213) 2.82 |  |
| **30.** | [**3COH**](http://www.rcsb.org/pdb/explore.do?structureId=3COH) | [**83H**](http://www.rcsb.org/pdb/ligand/ligandsummary.do?hetId=83H) | N7(83H) : O(A213) 2.83  N13(83H) : N(A213) 3.04 |  |
| **31.** | [**3E5A**](http://www.rcsb.org/pdb/explore.do?structureId=3E5A) | [**SO4**](http://www.rcsb.org/pdb/ligand/ligandsummary.do?hetId=SO4) |  |  |
|  |  | [**TPO**](http://www.rcsb.org/pdb/ligand/ligandsummary.do?hetId=TPO) |  |  |
|  |  | [**VX6**](http://www.rcsb.org/pdb/ligand/ligandsummary.do?hetId=VX6) | N14(VX6) : O(A213) 2.76  N20(VX6) : N(A213) 2.52 |  |
| **32.** | [**3EFW**](http://www.rcsb.org/pdb/explore.do?structureId=3EFW) | [**AK8**](http://www.rcsb.org/pdb/ligand/ligandsummary.do?hetId=AK8) | N2(AK8) : N(A213) 3.09  N4(AK8) : O(A213) 2.84  NZ(AK8) : O2(K162) 2.51 |  |
|  |  | [**SO4**](http://www.rcsb.org/pdb/ligand/ligandsummary.do?hetId=SO4) |  |  |
| **33.** | [**3FDN**](http://www.rcsb.org/pdb/explore.do?structureId=3FDN) | [**MMH**](http://www.rcsb.org/pdb/ligand/ligandsummary.do?hetId=MMH) | NAH(MMH) : O(A213) 2.92  NAB(MMH) : N(A213) 2.88  OBD(MMH) : N(T217) 3.17 |  |
| **34.** | [**3H0Y**](http://www.rcsb.org/pdb/explore.do?structureId=3H0Y) | [**48B**](http://www.rcsb.org/pdb/ligand/ligandsummary.do?hetId=48B) | N19(48B) : N(A213) 3.24  N24(48B) : O(A213) 2.88 |  |
|  |  | [**SO4**](http://www.rcsb.org/pdb/ligand/ligandsummary.do?hetId=SO4) |  |  |
| **35.** | [**3H0Z**](http://www.rcsb.org/pdb/explore.do?structureId=3H0Z) | [**45B**](http://www.rcsb.org/pdb/ligand/ligandsummary.do?hetId=45B) | CL1(45B) : OG1(T217) 3.26  N1(45B) : O(E260) 3.35  O1(45B) : N(T217) 3.32  N8(45B) : O(A213) 3.09 |  |
| **36.** | [**3H10**](http://www.rcsb.org/pdb/explore.do?structureId=3H10) | [**97B**](http://www.rcsb.org/pdb/ligand/ligandsummary.do?hetId=97B) | N4(97B) : O(A213) 2.71  N2(97B) : N(A213) 3.00 |  |
| **37.** | [**3HA6**](http://www.rcsb.org/pdb/explore.do?structureId=3HA6) | [**2JZ**](http://www.rcsb.org/pdb/ligand/ligandsummary.do?hetId=2JZ) | O37(2JZ) : NZ(K162) 2.92  O36(2JZ) : NZ(K162) 2.71  N29(2JZ) : N(A213) 3.15 |  |
|  |  | [**TPO**](http://www.rcsb.org/pdb/ligand/ligandsummary.do?hetId=TPO) |  |  |
| **38.** | [**3K5U**](http://www.rcsb.org/pdb/explore.do?structureId=3K5U) | [**PFQ**](http://www.rcsb.org/pdb/ligand/ligandsummary.do?hetId=PFQ) | N1(PFQ) : N(A213) 3.22 |  |
| **39.** | [**3LAU**](http://www.rcsb.org/pdb/explore.do?structureId=3LAU) | [**OFI**](http://www.rcsb.org/pdb/ligand/ligandsummary.do?hetId=OFI) | N6(OF1) : O(E211) 2.80  N7(OF1) : N(A213) 2.75  N8(OF1) : O(A213) 2.86 |  |
| **40.** | [**3MYG**](http://www.rcsb.org/pdb/explore.do?structureId=3MYG) | [**EML**](http://www.rcsb.org/pdb/ligand/ligandsummary.do?hetId=EML) | N2(EML) : N(A213) 3.00  N1(EML) : O(A213) 2.75  N8(EML) : NH2(R137) 3.32 |  |
|  |  | [**PG4**](http://www.rcsb.org/pdb/ligand/ligandsummary.do?hetId=PG4) |  |  |
| **41.** | [**3NRM**](http://www.rcsb.org/pdb/explore.do?structureId=3NRM) | [**NRM**](http://www.rcsb.org/pdb/ligand/ligandsummary.do?hetId=NRM) | N1(NRM) : O(A213) 2.72  N2(NRM) : N(A213) 2.92 |  |
| **42.** | [**3O50**](http://www.rcsb.org/pdb/explore.do?structureId=3O50) | [**LJE**](http://www.rcsb.org/pdb/ligand/ligandsummary.do?hetId=LJE) | O2(LJE) : NZ(K162) 3.07  N2(LJE) : N(A213) 2.82 |  |
| **43.** | [**3O51**](http://www.rcsb.org/pdb/explore.do?structureId=3O51) | [**LJF**](http://www.rcsb.org/pdb/ligand/ligandsummary.do?hetId=LJF) | N2(LJF) : N(A213) 2.90 |  |
| **1.** | [**2BFX**](http://www.rcsb.org/pdb/explore.do?structureId=2BFX) | [**TPO**](http://www.rcsb.org/pdb/ligand/ligandsummary.do?hetId=TPO) |  |  |
| **2.** | [**2BFY**](http://www.rcsb.org/pdb/explore.do?structureId=2BFY) | [**H1N**](http://www.rcsb.org/pdb/ligand/ligandsummary.do?hetId=H1N) | OBK(H1N) : N(A173) 2.85  NB1(H1N) : O(E171) 3.15  OAV(H1N) : NZ(K123) 2.62  OBE(H1N) : N(K103) 3.27 |  |
|  |  | [**TPO**](http://www.rcsb.org/pdb/ligand/ligandsummary.do?hetId=TPO) |  |  |
| **3.** | [**2VGO**](http://www.rcsb.org/pdb/explore.do?structureId=2VGO) | [**AD5**](http://www.rcsb.org/pdb/ligand/ligandsummary.do?hetId=AD5) | N9(AD5) : O(E171) 2.78  N3(AD5) : N(A173) 3.12  N2(AD5) : O(A173) 2.62 |  |
|  |  | [**TPO**](http://www.rcsb.org/pdb/ligand/ligandsummary.do?hetId=TPO) |  |  |
| **4.** | [**2VGP**](http://www.rcsb.org/pdb/explore.do?structureId=2VGP) | [**AD6**](http://www.rcsb.org/pdb/ligand/ligandsummary.do?hetId=AD6) | NAI(AD6) : N(A173) 2.95  NAK(AD6) : O(A173) 2.82  NAJ(AD6) : O(L99) 2.87 |  |
|  |  | [**TPO**](http://www.rcsb.org/pdb/ligand/ligandsummary.do?hetId=TPO) |  |  |
| **5.** | [**2VRX**](http://www.rcsb.org/pdb/explore.do?structureId=2VRX) | [**447**](http://www.rcsb.org/pdb/ligand/ligandsummary.do?hetId=447) | N3(447) : N(A213) 3.00 |  |
|  |  | [**TPO**](http://www.rcsb.org/pdb/ligand/ligandsummary.do?hetId=TPO) |  |  |
| **1.** | [**3D14**](http://www.rcsb.org/pdb/explore.do?structureId=3D14) | [**AK1**](http://www.rcsb.org/pdb/ligand/ligandsummary.do?hetId=AK1) | N6(AK1) : N(A226) 3.17  N13(AK1) : OE2 (E194) 2.96  N22(AK1) : OE1(E194) 2.78  O16(AK1) : NZ(K175) 3.11 |  |
| **2.** | [**3D15**](http://www.rcsb.org/pdb/explore.do?structureId=3D15) | [**AK2**](http://www.rcsb.org/pdb/ligand/ligandsummary.do?hetId=AK2) | O21(AK2) : NZ(K175) 2.66  N20(AK2) : OE1(E194) 2.72  N18(AK2) : OE2(E194) 2.86  N3(AK2) : N(A226) 3.06 |  |
| **3.** | [**3D2I**](http://www.rcsb.org/pdb/explore.do?structureId=3D2I) | [**AK3**](http://www.rcsb.org/pdb/ligand/ligandsummary.do?hetId=AK3) | N16(AK3) : N(A226) 2.96  N9(AK3) : OE2(E194) 2.93  N11(AK3) : OE1(E194) 2.71  O12(AK3) : NZ(L175) 3.29 |  |
| **4.** | [**3D2K**](http://www.rcsb.org/pdb/explore.do?structureId=3D2K) | [**AK4**](http://www.rcsb.org/pdb/ligand/ligandsummary.do?hetId=AK4) | N20(AK2) : OE1(E194) 2.72  N16(AK4) : N(A226) 2.89  N9(AK4) : OE2(E194) 2.89  O12(AK4) : NZ(K175) 2.73 |  |
| **5.** | [**3DAJ**](http://www.rcsb.org/pdb/explore.do?structureId=3DAJ) | [**FXG**](http://www.rcsb.org/pdb/ligand/ligandsummary.do?hetId=FXG) | O27(FXG) : N(F144) 2.74  N9(FXG) : O(E211) 2.90  O12(FXG) : N(E211) 2.99 |  |
| **6.** | [**3DJ5**](http://www.rcsb.org/pdb/explore.do?structureId=3DJ5) | [**AK5**](http://www.rcsb.org/pdb/ligand/ligandsummary.do?hetId=AK5) | O21(AK5) : NZ(K175) 2.75  O12(AK5) : NZ(K175) 3.24  N31(AK5) : N(A226) 2.99  N31(AK5) : 0(A226) 3.05 |  |
| **7.** | [**3DJ6**](http://www.rcsb.org/pdb/explore.do?structureId=3DJ6) | [**AK6**](http://www.rcsb.org/pdb/ligand/ligandsummary.do?hetId=AK6) | N31(AK6) : O(A226) 2.99  N27(AK6) : N(A226) 2.96  O6(AK6) : NZ(K175) 2.85  O15(AK6) : O15(K175) 3.16 |  |
| **8.** | [**3DJ7**](http://www.rcsb.org/pdb/explore.do?structureId=3DJ7) | [**AK7**](http://www.rcsb.org/pdb/ligand/ligandsummary.do?hetId=AK7) | N3(AK7) : N(A226) 2.99  N29(AK7) : O(A221) 3.08  N15(AK7) : OE2(E194) 2.94  N17(AK7) : OE1(E194) 2.81  O18(AK7) : NZ(K175) 2.83 |  |
